# Synthesis of uniform ordered mesoporous TiO_2_ microspheres with controllable phase junctions for efficient solar water splitting†
†Electronic supplementary information (ESI) available. See DOI: 10.1039/c8sc04155e


**DOI:** 10.1039/c8sc04155e

**Published:** 2018-11-21

**Authors:** Wei Zhang, Haili He, Yong Tian, Kun Lan, Qi Liu, Changyao Wang, Yang Liu, Ahmed Elzatahry, Renchao Che, Wei Li, Dongyuan Zhao

**Affiliations:** a Department of Chemistry , State Key Laboratory of Molecular Engineering of Polymers , Shanghai Key Lab of Molecular Catalysis and Innovative Materials , Laboratory of Advanced Materials , iChEM , Fudan University , Shanghai 200433 , P. R. China . Email: dyzhao@fudan.edu.cn ; Email: weilichem@fudan.edu.cn; b Materials Science and Technology Program , College of Arts and Sciences , Qatar University , PO Box 2713 , Doha 2713 , Qatar

## Abstract

As a benchmark photocatalyst, commercial P25–TiO_2_ has been widely used for various photocatalytic applications. However, the low surface area and poorly porous structure greatly limit its performance. Herein, uniform ordered mesoporous TiO_2_ microspheres (denoted as Meso-TiO_2_-*X*; *X* represents the rutile percentage in the resultant microspheres) with controllable anatase/rutile phase junctions and radially oriented mesochannels are synthesized by a coordination-mediated self-assembly approach. The anatase/rutile ratio in the resultant microspheres can be facilely adjusted as desired (rutile percentage: 0–100) by changing the concentration of hydrochloric acid. As a typical one, the as-prepared Meso-TiO_2_-25 microspheres have a similar anatase/rutile ratio to commercial P25. But the surface area (78.6 m^2^ g^–1^) and pore volume (0.39 cm^3^ g^–1^) of the resultant microspheres are larger than those of commercial P25. When used as the photocatalyst for H_2_ generation, the Meso-TiO_2_-25 delivers high solar-driven H_2_ evolution rates under air mass 1.5 global (AM 1.5 G) and visible-light (*λ* > 400 nm), respectively, which are significantly larger than those of commercial P25. This coordination-mediated self-assembly method paves a new way toward the design and synthesis of high performance mesoporous photocatalysts.

## Abstract 2

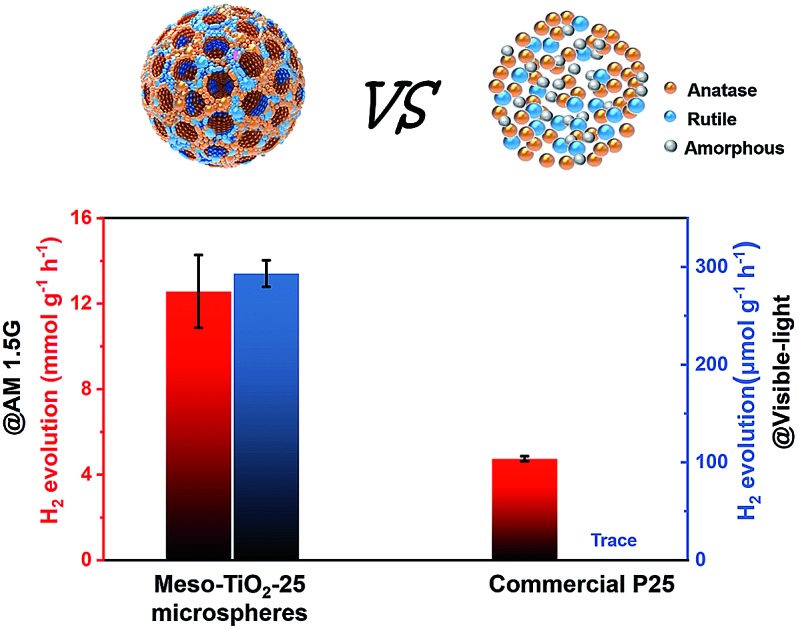
Mesoporous TiO_2_ microspheres with controllable phase junctions have been synthesized by a facile coordination-mediated self-assembly method.

## Introduction

Since the first report in 1972 by Fujishima and Honda,^1^ TiO_2_ has become one of the most widely investigated photocatalysts for various applications because of its low cost, non-toxicity, chemical stability and high resistance to photocorrosion.^2^–^11^ However, the photocatalytic efficiency of TiO_2_ materials has been greatly limited by their wide band gap (3.0–3.2 eV) and low quantum efficiency that results from the fast recombination of photo-generated electrons and holes.^12^–^14^ To date, tremendous efforts have been devoted to overcoming these problems to improve photocatalytic activities.^15^–^19^ Despite great progress achieved recently, it is still difficult to prepare TiO_2_ materials with higher photocatalytic activities than commercial P25–TiO_2_, which is widely used as a benchmark photocatalyst. It has been demonstrated that the good performance of commercial P25 originates from its partially contacting anatase and rutile nanoparticles.^20^,^21^ The transfer of photoexcited electrons at the anatase–rutile interface enhances charge separation and then produces a high photocatalytic performance.^22^ Therefore, coupling of anatase and rutile particles to form phase junctions is fast becoming a strategy to improve the photocatalytic activity of TiO_2_. However, commercial P25 is produced by a vapor-fed flame aerosol technology, which is complex and requires special equipment; moreover, it is hard or impossible to control the phase ratio and nanostructures.^23^ Besides, the amorphous TiO_2_ nanoparticles in commercial P25 have a negative effect on photocatalytic performance.^24^ Later, a one-step phase transformation method at a high temperature^25^,^26^ and two-step assembly method^27^–^29^ with pre-prepared anatase or rutile building blocks have been developed toward constructing highly efficient anatase–rutile phase junctions. Nevertheless, it is still a great challenge to facilely synthesize highly crystalline TiO_2_ materials with phase junctions and porous structures.

Surface sites and structures are another critical issue for photocatalysts because the reaction takes place only when photoinduced electrons and holes are available on the surface.^30^ Recently, various kinds of TiO_2_ nanostructures have been synthesized to increase surface-active sites and adjust interfacial chemistries, like TiO_2_ single crystals,^31^,^32^ TiO_2_ mesocrystals,^33^–^35^ heteroatom-doped TiO_2_ (ref. 16), black TiO_2_ (ref. 18) *etc.* Among them, mesoporous TiO_2_ materials are of particular interest due to their high surface areas, and large pore sizes and volumes. This not only increases the density of active sites with high accessibility, but also facilitates the diffusion of reactants and products.^36^–^39^ Therefore, the low surface areas (<50 m^2^ g^–1^) and poorly porous structures of the commercial P25, even for the synthesized TiO_2_ materials, significantly limit their performances. Up to now, considerable efforts have been made toward synthesizing mesoporous TiO_2_ materials through various approaches.^40^–^44^ However, these methods usually give rise to products with ill-defined mesostructures, poor crystallinity and low porosity, which is unfavorable for improving the photocatalytic performances. Moreover, to the best of our knowledge, there is no report about synthesis of mesoporous TiO_2_ materials with controllable anatase–rutile ratios, intimately contacting phase junctions and high crystallinity.

Herein, we demonstrate a new facile coordination-mediated self-assembly strategy to synthesize Meso-TiO_2_-25 by using Pluronic F127 as the template, tetrabutyl titanate (TBOT) as the precursor and hydrochloric acid (HCl) as the coordination agent for mediating the coordination modes of Ti^4+^ ions. Besides, acetic acid (HOAc) is utilized so that the TBOT can effectively match the cooperative assembly with Pluronic F127. The resultant Meso-TiO_2_-25 with a uniform spherical morphology is composed of highly crystalline anatase and rutile nanoparticles with a size of ∼15 nm, and the anatase/rutile ratio is measured to be ∼77 : 23, which is similar to that of commercial P25 (∼79 : 21). But the Meso-TiO_2_-25 has a much higher surface area (78.6 m^2^ g^–1^) and larger pore volume (0.39 m^2^ g^–1^) than commercial P25. More importantly, the anatase/rutile ratio can be well adjusted (rutile percentage: 0–100) by changing the concentration of HCl. When used as the photocatalyst, the Meso-TiO_2_-25 shows excellent photocatalytic performance, including high hydrogen evolution rates under AM 1.5 G and visible-light, respectively, which are considerably larger than those of commercial P25.

## Results and discussion

**Fig. 1:**
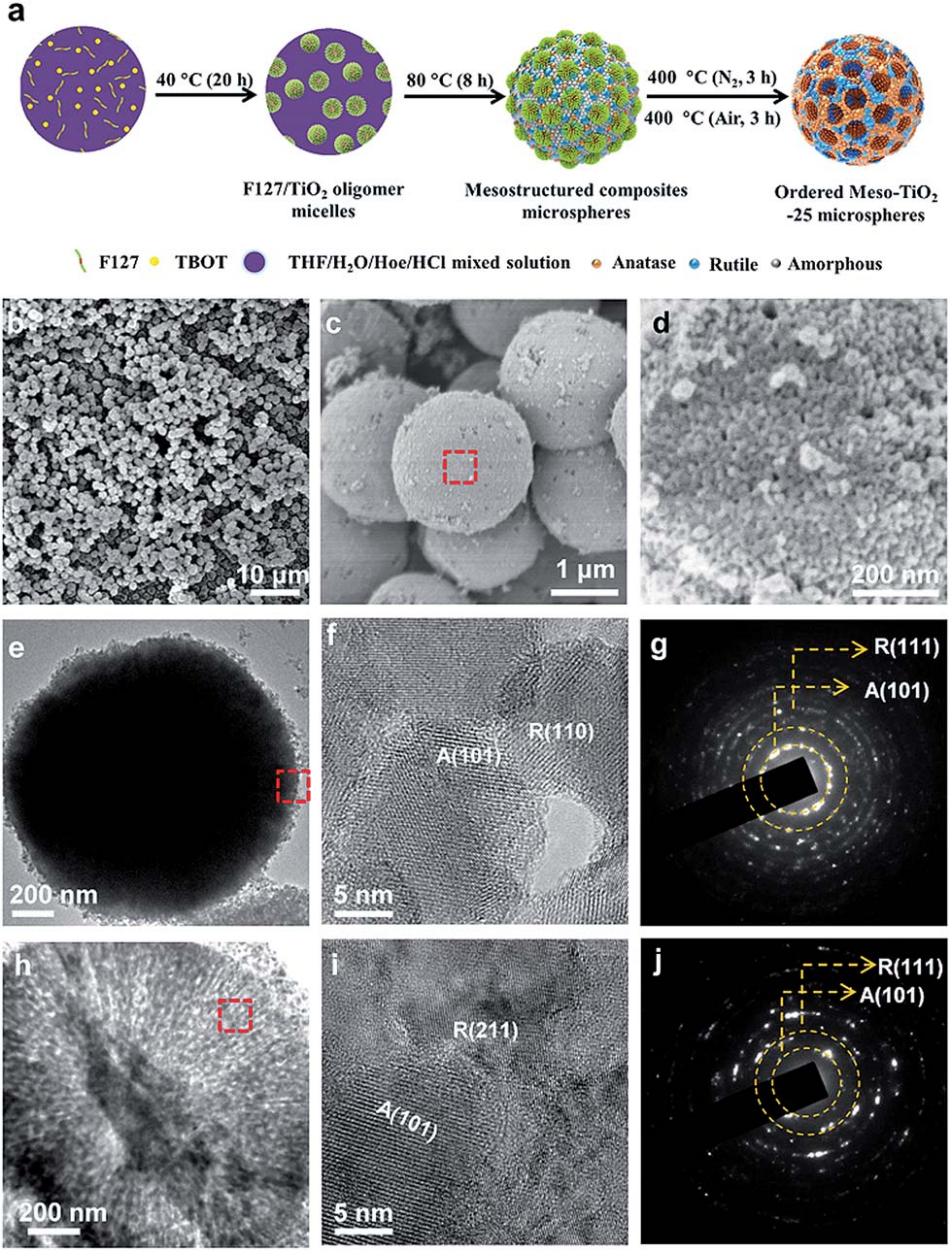
(a) Schematic illustration of the synthesis process of Meso-TiO_2_-25 microspheres *via* a facile coordination-mediated self-assembly strategy. (b–d) FESEM, (e and h) TEM, and (f and i) HRTEM images, and (g and j) SAED patterns of the Meso-TiO_2_-25 prepared by the coordination-mediated self-assembly method. Images (f and g) and (i and j) were taken from the surface and inside domain, respectively, revealing the polycrystallinity of mixed anatase and rutile TiO_2_ nanoparticles. (d), (f) and (i) are enlarged images of the domains of the red rectangle in (c), (e) and (h), respectively.

The synthesis procedure of the Meso-TiO_2_-25 is illustrated in Fig. 1a. First, Pluronic F127 and TBOT were dissolved in a THF solution with the assistance of HCl and HOAc to form a homogeneous solution. Next, the solution was placed in an oven at 40 °C for evaporation; as a result, uniform spherical F127/TiO_2_ oligomer composite micelles could be formed. Subsequent slow evaporation at 80 °C resulted in the micellar self-assembly and structural transformation, leading to the formation of ordered mesostructured composite microspheres. During this process, the coordination modes of Ti^4+^ ions could be mediated by the coordination agent in the synthetic system, and then TiO_2_ nanocrystals with different crystal phases are formed. Finally, the Meso-TiO_2_-25 could be obtained by calcination of the mesostructured composite microspheres in N_2_ and air, respectively.

Field-emission scanning electron microscopy (FESEM) images clearly show that all the obtained Meso-TiO_2_-25 microspheres have a uniform spherical morphology with a mean diameter of ∼1.8 μm (Fig. 1b and c). Cylindrical open mesopores with a size of ∼8 nm exposed on the rough surface can be observed. A magnified FESEM image (Fig. 1d) shows that the Meso-TiO_2_-25 consists of numerous nanoparticles with a size of ∼15 nm, which is smaller than that of commercial P25 (∼25 nm, Fig. S1a, ESI†). The transmission electron microscopy (TEM) images (Fig. 1e) of the Meso-TiO_2_-25 further reveal its well-defined spherical shape with a diameter of ∼1.8 μm. The high-resolution TEM (HRTEM) images recorded from the surface of a single Meso-TiO_2_-25 microsphere show that the nanoparticles are highly crystallized and interconnected with a size of ∼15 nm. The interplanar distances are measured to be ∼0.35 and 0.32 nm, which are well matched with the *d*_101_ spacing of anatase and *d*_110_ spacing of rutile, respectively (Fig. 1f). The selected-area electron diffraction (SAED) pattern (Fig. 1g) taken from the corresponding part shows two series of well-defined diffraction patterns, which can be assigned to polycrystalline anatase and rutile, respectively, suggesting the formation of anatase–rutile phase junctions. To analyse the internal structure, the Meso-TiO_2_-25 was embedded in a resin and cut into slices with a microtome for TEM characterization. The cylindrical mesopores are distributed radially from the centre to the surface along the restricted tangential direction with a uniform pore size of ∼8 nm (Fig. 1h), which is much different from that of the commercial P25 composed of irregular nanoparticles with a poorly porous structure (Fig. S1b, ESI†). The inner pore walls are composed of many intimately contacting nanoparticles, which are also highly crystallized (Fig. 1i). The corresponding SAED image (Fig. 1j) taken from the inner part further confirms the formation of anatase–rutile phase junctions. In addition, three representative interfacial domains of one single Meso-TiO_2_-25 microsphere from outside to inside were selected to analyze the connection of anatase–rutile phases (Fig. S2, ESI†). It is clearly seen that anatase and rutile nanoparticles have high crystallinity and are directly connected without any other phase or/and amorphous matter. The commercial P25 is obviously distinguished with partially amorphous connection (Fig. S1c and S1d, ESI†).^21^,^24^ The direct connection can be further confirmed by a large number of randomly selected HRTEM images (Fig. S3, ESI†). According to statistic results, the exposed crystal faces of anatase and rutile nanoparticles are randomly oriented, but most of them have a good lattice-matching relationship (Fig. S4, ESI†) between each other.

**Fig. 2:**
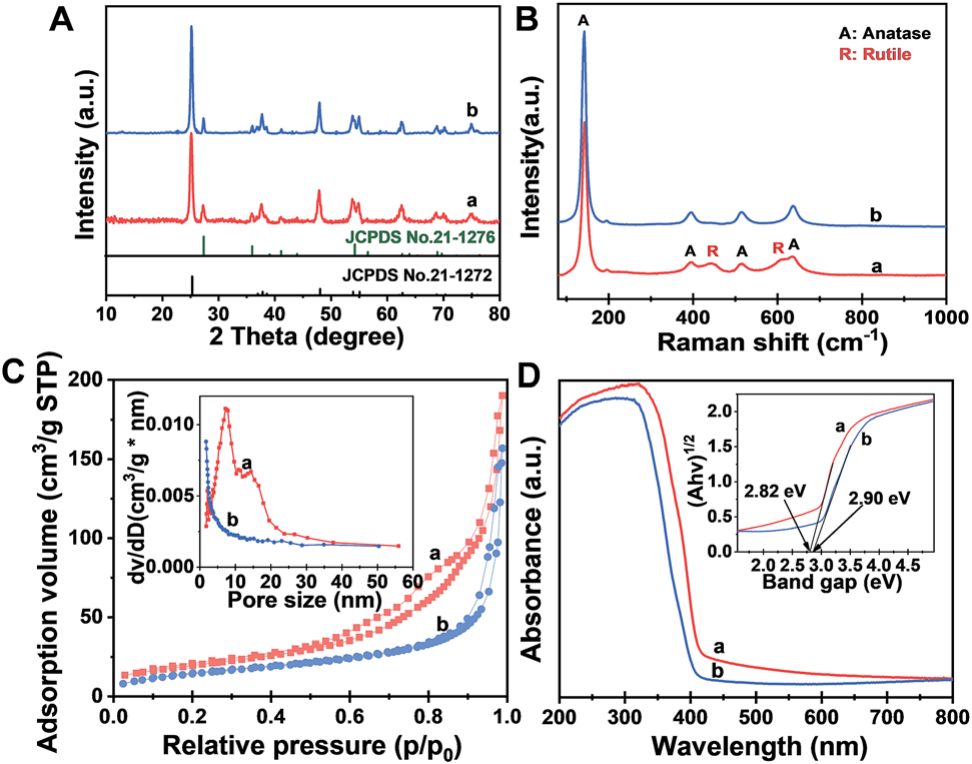
(A) XRD patterns, (B) Raman spectra, (C) nitrogen adsorption–desorption isotherms and (D) UV-Vis diffuse reflectance spectra of (a) the Meso-TiO_2_-25 and (b) commercial P25. The insets in (C) and (D) are the corresponding pore size distribution curves and Kubelka–Munk plots transformed from UV-Vis diffuse reflectance spectra, respectively.

The X-ray diffraction (XRD) pattern (Fig. 2A) of the Meso-TiO_2_-25 exhibits two series of diffraction peaks, which can be indexed to the anatase (JCPDS no. 21-1272) and rutile (JCPDS no. 21-1276) phases, respectively. The anatase/rutile ratio is measured to be ∼77 : 23, which is similar to that of the commercial P25 (∼79 : 21). The average crystal size of the Meso-TiO_2_-25 microspheres can be calculated to be ∼14 nm by using the Scherrer equation, which is smaller than that of commercial P25 (∼21 nm). The Raman spectrum (Fig. 2B) of the Meso-TiO_2_-25 microspheres shows four well-defined bands at 144, 396, 515, and 637 cm^–1^, which can be assigned to the typical vibrational modes of the anatase phase. Moreover, the typical vibrational modes of the rutile phase can also be distinguished from the intense bands at 445 and 610 cm^–1^, indicating a mixed phase structure on the surface. In contrast, the Raman spectrum of the commercial P25 exhibits a typical anatase phase, which may have resulted from the rutile phase generally existing in the interior of the bulk phase.^45^

The nitrogen adsorption–desorption isotherms of the Meso-TiO_2_-25 (Fig. 2C) show characteristic type IV curves with two capillary condensation steps, indicating bimodal mesopore distributions. A distinct capillary condensation step at *P*/*P*_0_ = 0.50 to 0.80 reflected uniform cylindrical mesopores resulting from the primary radial mesochannels, which are templated with Pluronic F127. Moreover, a hysteresis loop at a higher pressure (*P*/*P*_0_ = 0.90 to 0.99) reflected the structural defects formed during the pyrolysis. However, the commercial P25 shows typical sorption isotherms resulting from nanoparticle aggregation. The Brunauer–Emmett–Teller (BET) surface area and pore volume of the Meso-TiO_2_-25 are calculated to be as high as 78.4 m^2^ g^–1^ and 0.39 cm^3^ g^–1^, respectively, which are much higher than those of the commercial P25 (Table 1). The pore size (the inset in Fig. 2C) of the Meso-TiO_2_-25 calculated by using the Barrett–Joyner–Halenda (BJH) model reveals the presence of two sets of pores with diameters centered at 7.5 and 15.6 nm, respectively, which are significantly larger than that of the commercial TiO_2_ (<2 nm). The UV-Vis spectrum (Fig. 2D) of the Meso-TiO_2_-25 clearly shows a broad absorption starting at 420 nm in the ultraviolet region, which has a red shift compared with that of the commercial P25 starting at 400 nm. According to the Kubelka–Munk equation,^46^ the band gap of the Meso-TiO_2_-25 is calculated to be about 2.82 eV (the inset in Fig. 2D), which is smaller than the 2.90 eV of the commercial P25. The narrowing band gap of the Meso-TiO_2_-25 can be further confirmed by a blue shift of the valence band maximum energy (Fig. S5, ESI†), which may have resulted from the formation of surface defects during the sol–gel process (Fig. S6 and S7, ESI†).^47^ X-ray photoelectron spectroscopy (XPS) spectra of the Meso-TiO_2_-25 and commercial P25 are identical (Fig. S8 and S9, ESI†), suggesting their similar composition without any impurities.

**Table 1:** Structural and textural properties of the TiO_2_ photocatalysts prepared under different conditions

	Surface area^*a*^ (m^2^ g^–1^)	Pore volume^*b*^ (cm^3^ g^–1^)	Composition^*c*^	Average particle size^*d*^ (nm)
Meso-TiO_2_-0	90.2	0.29	Anatase	12.1
Meso-TiO_2_-25	78.4	0.39	Anatase : rutile = 77 : 23	13.5
Meso-TiO_2_-40	70.5	0.38	Anatase : rutile = 58 : 42	13.3
Meso-TiO_2_-60	63.6	0.28	Anatase : rutile = 40 : 60	14.6
Meso-TiO_2_-100	52.2	0.17	Rutile	15.3
Commercial P25	43.8	0.24	Anatase : rutile = 79 : 21	20.6
Nonporous-TiO_2_-25	15.6	0.06	Anatase : rutile = 77 : 23	17.8

^*a*^BET specific surface areas calculated using nitrogen adsorption–desorption isotherms.

^*b*^Total pore volumes estimated based on the volume adsorbed at a *P*/*P*_0_ of 0.995.

^*c*^The ratio of anatase/rutile estimated from XRD patterns.

^*d*^The average crystal size estimated using the Scherrer equation from XRD patterns.

**Fig. 3:**
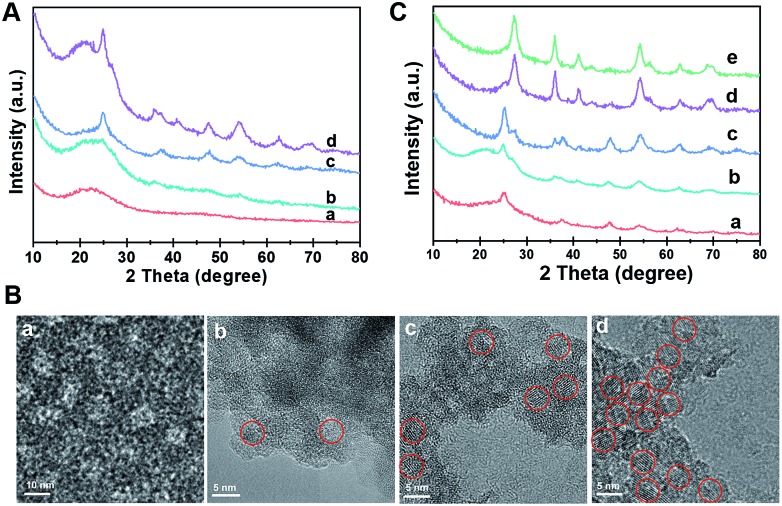
(A) XRD patterns and (B) HRTEM images of the intermediate products directly taken for different reaction times during the evaporation process: (a) 40 °C, for 20 h, (b) 80 °C, for 2 h, (c) 80 °C, for 4 h, and (d) 80 °C, for 8 h. The TiO_2_ nanocrystals are highlighted by red circles. (C) XRD patterns of the ordered mesoporous TiO_2_ microspheres synthesized by the seed-mediated self-assembly method through changing the concentration of HCl: (a) 0.3 M, (b) 0.6 M, (c) 0.9 M, (d) 1.2 M and (e) 1.5 M.

The formation process of the Meso-TiO_2_-25 was monitored by the *ex situ* XRD and *ex situ* TEM measurements. After evaporation at 40 °C for 20 h, a gel-like solution mixture can be obtained (Fig. S10, ESI†), which contains F127/TiO_2_ oligomer composite micelles with a diameter of ∼10 nm. When being dried on the TEM grid, they can self-assemble into an ordered arrangement (Fig. 3A and B). On further evaporation at 80 °C for 2 h, some precipitates are formed at the bottom of the vial due to the self-assembly of the micelles and the quick condensation of the titanium species. Notably, some anatase seeds can be distinguished in the amorphous mesostructured matrix. After 4 h at 80 °C, the mixture is almost dry and the amount of anatase nanocrystals in the mesostructures dramatically increases. On further aging at 80 °C for a long time, the grain size of the nanocrystals continuously grows. Interestingly, a small number of rutile seeds are formed in the resultant mesostructures. After pyrolysis at 400 °C, the XRD patterns show that highly crystallized ordered mesoporous anatase and/or rutile structures can be obtained (Fig. S11, ESI†). Without using Pluronic F127, irregular TiO_2_ nanoparticles can be obtained, and these particles have a similar polymorph structure to the Meso-TiO_2_-25 (Fig. S12, ESI†). In addition, without using HOAc, similar irregular TiO_2_ nanoparticles are obtained, indicating that HOAc can make the hydrolysis of TBOT more controllable and then effectively match the cooperative assembly with Pluronic F127 (Fig. S13, ESI†). Moreover, the XRD patterns show that HOAc has a minor effect on the crystal phase (Fig. S14, ESI†).

The effect of HCl on the formation of TiO_2_ polymorphs in the mesopore walls was investigated (Fig. 3C). When a small fraction of HCl is used, even after evaporation at 80 °C for 8 h, only anatase can be detected in the resultant mesostructured samples. As the amount of HCl increases to a high level, a large number of anatase nanoparticles are formed, and meanwhile, some rutile nanoparticles can also be distinguished. Gradually, the as-prepared microspheres composed of pure rutile can be obtained on further increasing the concentration of HCl. After calcination, highly crystalline ordered mesoporous TiO_2_ microspheres with controllable anatase/rutile ratios are obtained (Fig. S15 and S16, ESI†). The resultant ordered mesoporous TiO_2_ microspheres with different anatase/rutile ratios are high crystallized and show uniform spherical morphologies (Fig. S17, ESI†), which are similar to the Meso-TiO_2_-25.

Here, we propose a coordination-mediated self-assembly strategy for the synthesis of Meso-TiO_2_-25. In this case, the hydrolysis and condensation processes of TBOT are well adjusted with HCl, leading to the formation of titanium oligomers. In the presence of Pluronic F127, the resultant oligomers can effectively interact with the PEO chains through hydrogen bonding with the assistance of HOAc during the evaporation process and then uniform spherical F127/titanium oligomer micelles can be formed. A continuous slow evaporation of residual THF and the hydrolyzed solvent triggers the fusion of the initially formed spherical micelles into radially oriented cylindrical micelles, and then mesostructured microspheres can subsequently be obtained.^48^ At the same time, further condensation of the titanium oligomers leads to the nucleation and growth of anatase and rutile, which is significantly influenced by the coordination modes of Ti^4+^ ions.

In this system, Ti^4+^ ions are coordinated with –OH groups and Cl^–^ ions due to the hydrolysis of TBOT with the assistance of HCl thus causing the formation of partially hydrolyzed [Ti(OH)_*n*_Cl_*m*_]^2–^ (*n* + *m* = 6) octahedra. The nucleation and growth processes of anatase and rutile occur through the dehydration and cross-linking between [Ti(OH)_*n*_Cl_*m*_]^2–^ octahedra.^49^ Edge-shared bonding with two dehydration reactions and corner-shared bonding with one dehydration reaction are the two-major cross-linking ways between [Ti(OH)_*n*_Cl_*m*_]^2–^ octahedra. In the rutile structure, each octahedron is surrounded by ten octahedra, of which two of them are edge-shared and eight are corner-shared while in the anatase structure, each octahedron is surrounded by eight octahedra with four being edge-shared and four corner-shared.^50^ This means that the cross-linking of [Ti(OH)_*n*_Cl_*m*_]^2–^ octahedra with more edge-shared octahedra favors the nucleation and growth processes of anatase, while the cross-linking of [Ti(OH)_*n*_Cl_*m*_]^2–^ octahedra with more corner-shared octahedra can promote the nucleation and growth of rutile.

**Scheme 1:**
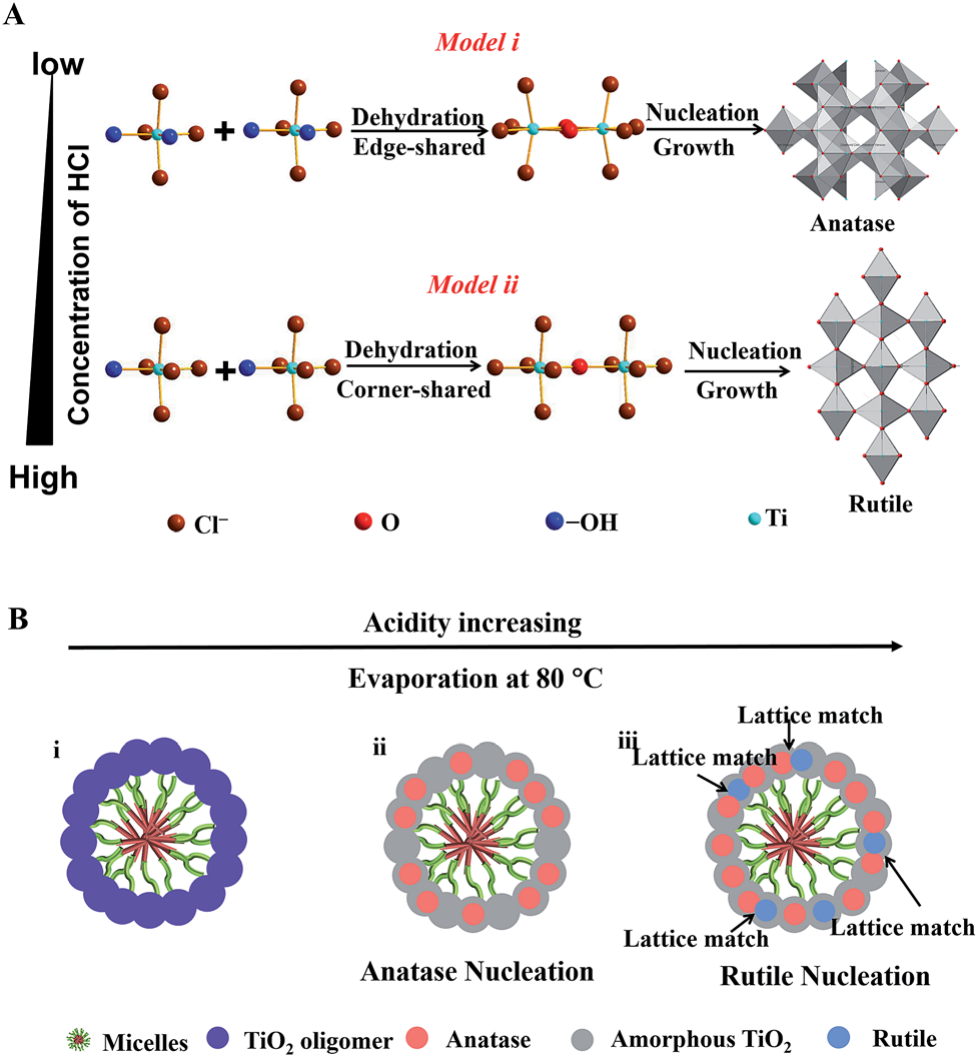
(A) Schematic representation of the process of TiO_2_ nucleation and cross-linking of [Ti(OH)_*n*_Cl_*m*_]^2–^ octahedra during the solvent evaporation. There are two distinct cross-linking ways of [Ti(OH)_*n*_Cl_*m*_]^2–^ octahedra under the different coordination conditions. (B) Schematic illustration of the nucleation and growth processes of TiO_2_ nanocrystals during assembly with the amphiphilic triblock copolymer micelles *via* the coordination-mediated self-assembly method.

At a low concentration of HCl, the hydrolysis degree of TBOT is relatively high and the concentration of Cl^–^ in the solution is rather low; thus the coordination number of –OH (*n*) in [Ti(OH)_*n*_Cl_*m*_]^2–^ is high. As a result, the probability of two dehydration reactions is larger, which favor the nucleation and growth of the anatase phase with more edge-shared [Ti(OH)_*n*_Cl_*m*_]^2–^ octahedra (Scheme 1A, model i). In contrast, the high concentration of HCl in the feedstock can greatly inhibit the hydrolysis and the concentration of Cl^–^ is high; thus the coordination of –OH groups (*n*) in [Ti(OH)_*n*_Cl_*m*_]^2–^ is low. Hence, the corner-shared process with one dehydration reaction between [Ti(OH)_*n*_Cl_*m*_]^2–^ octahedra occurs more easily, which is beneficial for the nucleation and growth of rutile (Scheme 1A, model ii). In the synthesis of the Meso-TiO_2_-25, the nucleation and growth of anatase occur first due to the low concentration of HCl initially. With the solvent evaporation, the concentration of HCl can be increased in the synthesis system, and the nucleation and growth of the rutile phase begin (Scheme 1B).

During the nucleation and growth, the resultant anatase and rutile nanocrystals are randomly oriented, but most of them have a lattice-matching relationship,^51^ such as the facet (101) of anatase (*d*_spacing_ = 0.35 nm) and the facet (110) of rutile (*d*_spacing_ = 0.32 nm). The lattice-matched anatase and rutile nanocrystals can come into contact easily by the confinement effect of Pluronic F127. After calcination at 400 °C, Pluronic F127 templates are removed and the mesopore walls are fully crystallized. During this process, the resultant anatase and rutile nanocrystals can further grow. Therefore, the intensity of the diffraction peaks attributed to the anatase and rutile phases increases after annealing. With the increase of pyrolysis temperature, the sizes of the anatase and rutile nanoparticles grow and the percentage of rutile in the resulting microspheres increases gradually, which are consistent with the results reported previously (Fig. S18, ESI†).^25^ At the same time, the ordered porous structure of the resulting microspheres cannot be retained after annealing at a high temperature (Fig. S19, ESI†). Therefore, we choose 400 °C as the final pyrolysis temperature because the templates can be removed completely, and the ordered porous structure can be retained at this temperature. Finally, Meso-TiO_2_-25 composed of intimately contacting anatase and rutile nanoparticles of uniform size can be obtained. Moreover, sub-kilogram scale Meso-TiO_2_-25 of uniform diameter can be prepared in one-pot (∼10 L) with a high yield of ∼84% (Fig. S20, ESI†), indicating that this synthetic method has the potential to be scalable for mass production and practical applications.

**Fig. 4:**
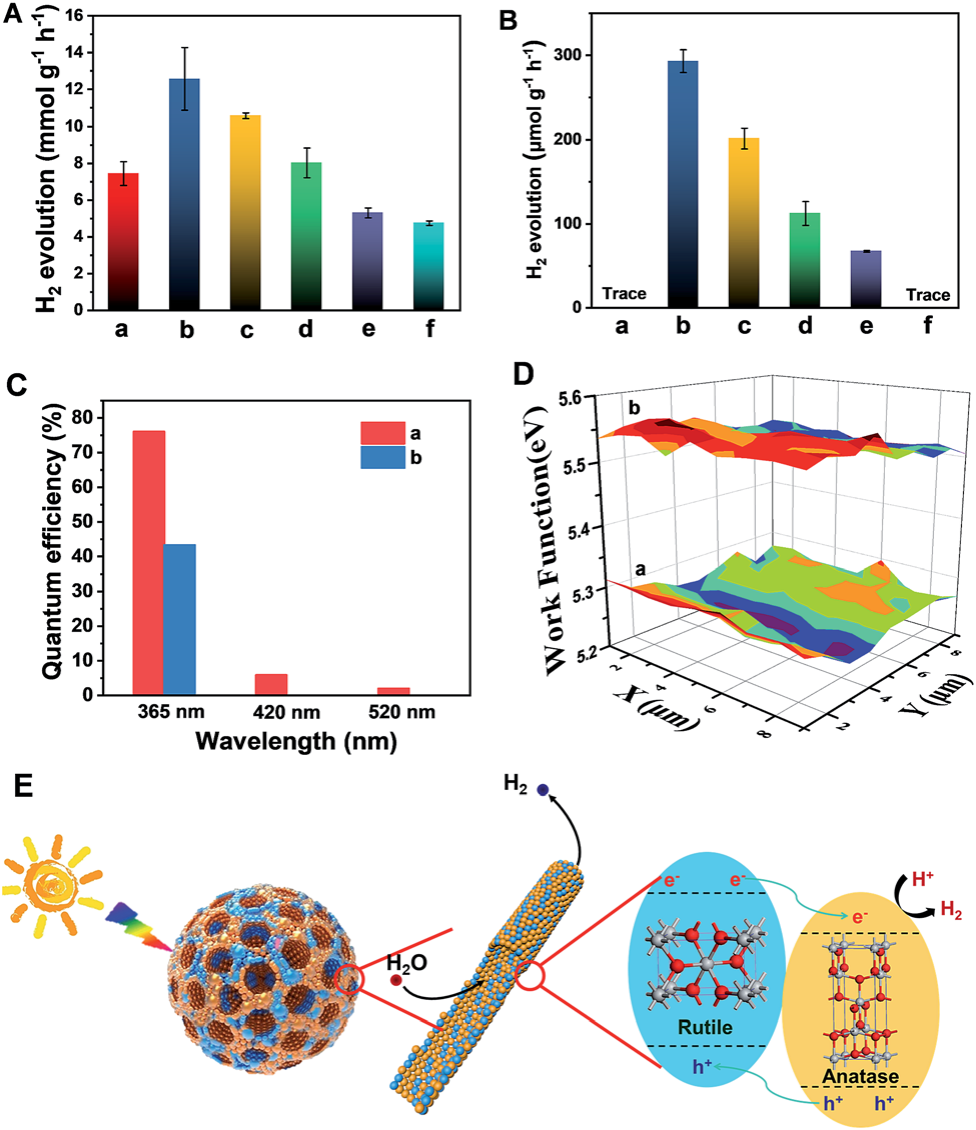
H_2_ evolution rates under (A) AM 1.5 G and (B) visible-light (*λ* > 400 nm) of the (a) Meso-TiO_2_-0, (b) Meso-TiO_2_-25, (c) Meso-TiO_2_-40 (d) Meso-TiO_2_-60, and (e) Meso-TiO_2_-100 microspheres, and (f) commercial P25. The (C) quantum efficiencies and (D) surface work functions of the (a) Meso-TiO_2_-25 microspheres and (b) commercial P25 under single-wavelength light. 50 mg of the photocatalysts loaded with 1% wt Pt as the co-catalyst were used for all experiments in a 25 vol% aqueous methanol solution. (E) Schematic diagram illustrating the process of H_2_ evolution across the Meso-TiO_2_-25.

The resultant Meso-TiO_2_-25 was evaluated as a H_2_ evolution photocatalyst with Pt as the co-catalyst. Commercial P25, Meso-TiO_2_-0, Meso-TiO_2_-40, Meso-TiO_2_-60, and Meso-TiO_2_-100 microspheres were used as the control samples. The Meso-TiO_2_-25 microspheres show a photocatalytic hydrogen evolution rate of 12.6 mmol h^–1^ g^–1^ under AM 1.5 G (Fig. 4A), which is much higher than those of the commercial P25 (4.74 mmol h^–1^ g^–1^), Meso-TiO_2_-0 (7.44 mmol h^–1^ g^–1^), Meso-TiO_2_-40 (10.6 mmol h^–1^ g^–1^), Meso-TiO_2_-60 (8.02 mmol h^–1^ g^–1^) and Meso-TiO_2_-100 (5.30 mmol h^–1^ g^–1^) microspheres. The highest hydrogen evolution rate of Meso-TiO_2_-25 can be attributed to its high surface area and highly crystalline anatase/rutile phase junctions (Table 1). Furthermore, we have also measured photocatalytic H_2_ evolution performances by cutting off the UV light shorter than 400 nm (Fig. 4B). The H_2_ generation rate of the Meso-TiO_2_-25 microspheres is as high as 293 μmol g^–1^ h^–1^, which is significantly larger than that of the other samples. It should be noted that this value is even comparable to those of the best black TiO_2_-based photocatalysts reported previously (Table S1, ESI†). Besides, no noticeable decrease in the H_2_ production rate for the Meso-TiO_2_-25 is observed in the four cycling tests within 12 h under AM 1.5 G and visible-light (Fig. S21, ESI†), respectively, indicating its high photostability. Significantly, the apparent quantum efficiency (Fig. 4C) and the corresponding H_2_ generation rate of the Meso-TiO_2_-25 under 365 nm are measured to be 76.1% and 151 μmol h^–1^ (Fig. S22, ESI†), respectively. These values are significantly higher than those of commercial P25 (43.3% and 85.9 μmol h^–1^). Besides, the quantum efficiencies of the Meso-TiO_2_-25 microspheres can reach 5.9 and 2.0% under light of 420 and 520 nm, respectively, with the corresponding H_2_ generation rates as high as 4.0 and 1.6 μmol h^–1^. In contrast, the commercial P25 shows no obvious visible-light activity. Furthermore, the photocatalytic performances of Meso-TiO_2_-25 and commercial P25 for decomposition of Rhodamine B (RhB) were investigated without using noble metals as cocatalysts (Fig. S23, ESI†). The photocatalytic decomposition of RhB (1 × 10^–4^ mol L^–1^) over Meso-TiO_2_-25 could be completed in 40 min under simulated sunlight irradiation, which is faster than the 85 min for the commercial P25.

The linear sweep voltammogram of the Meso-TiO_2_-25 shows the highest photocurrent density of ∼0.70 mA cm^–2^ and the lowest saturation potential of –0.7 V *vs.* Ag/AgCl among all samples (Fig. S24, ESI†), implying that the charge separation and transportation are more efficient.^52^ The time-dependent photocurrent measurements of the Meso-TiO_2_-25 show a stable photocurrent density and an excellent correlation with the on/off cycles of simulated solar light (Fig. S25, ESI†). This photocurrent is highly stable under continuous solar illumination for 5000 s (Fig. S26, ESI†), indicating the excellent stability for long-term PEC conversion. The fluorescence emission spectra of the samples are similar in shape. Two emission peaks at 421 and 400 nm are observed, which can be attributed to the emission of the band gap transition of rutile (3.0 eV) and anatase (3.2 eV), respectively.^53^ The weakest fluorescence intensity and the longest decay time (Fig. S27, ESI†) of Meso-TiO_2_-25 indicate the long lifetime of the photogenerated charges and holes, further confirming the efficient charge separation. The lower surface work function of the Meso-TiO_2_-25 (5.28 eV) ensures easier escape of electrons from the surface and then to the co-catalysts for hydrogen evolution (Fig. 4D). As a result, the electron–hole recombination can be reduced and the photocatalytic performance can be improved.

The excellent photocatalytic performances can be majorly attributed to the unique nanostructures (Fig. 4E): (i) the radially mesoporous structure with a large pore size can shorten the electrolyte transport pathway, promoting the photocatalytic reaction kinetics; (ii) the high surface areas can provide more exposed active sites for surface reduction reactions; (iii) the surface defects can lower the band gap to some extent, thus greatly enhancing the visible light absorption; (iv) the intimately contacting anatase–rutile phase junctions and highly crystalline anatase and rutile nanoparticles in the pore walls can improve the charge separation and transport, thereby increasing the quantum efficiency.

## Conclusions

In summary, we demonstrate a coordination-mediated self-assembly strategy for the preparation of Meso-TiO_2_-25 with the ratio of anatase–rutile similar to that of commercial P25 for the first time. The as-prepared Meso-TiO_2_-25 has a large pore volume (0.39 cm^3^ g^–1^) and high surface area (78.6 m^2^ g^–1^), which are almost two times higher than those of the commercial P25. More importantly, the ratio of anatase/rutile can be easily adjusted (rutile percentage: 0–100) by changing the concentration of HCl. The unique mesoporous structure and intimately contacting and highly crystalline anatase–rutile phase junctions make the resultant Meso-TiO_2_-25 ideal for photoelectrochemical conversion and photocatalytic water splitting. It shows a high hydrogen production rate and photocurrent density, which are ∼3 and ∼2 times as high as those of the commercial P25, respectively. Significantly, the Meso-TiO_2_-25 shows excellent visible-light activity and its H_2_ evolution rate is as high as 293 μmol g^–1^ h^–1^ under the light of *λ* > 400 nm, while the commercial P25 shows no sign of visible-light activity. This work opens an avenue for rational synthesis of semiconductor photocatalysts with mesoporous and controllable phase junctions for various applications.

## Conflicts of interest

There are no conflicts to declare.

## Supplementary Material

Supplementary information

## References

[cit1] Fujishima A., Honda K. (1972). Nature.

[cit2] Ma Y., Wang X., Jia Y., Chen X., Han H., Li C. (2014). Chem. Rev..

[cit3] Liu G., Yang H. G., Pan J., Yang Y. Q., Lu G. Q., Cheng H.-M. (2014). Chem. Rev..

[cit4] Hirakawa H., Hashimoto M., Shiraishi Y., Hirai T. (2017). J. Am. Chem. Soc..

[cit5] Liu Y., Luo Y., Elzatahry A., Luo W., Che R., Fan J., Lan K., Al-Enizi A., Sun Z., Li B., Liu Z., Shen D., Ling Y., Wang C., Wang J., Gao W., Yao C., Yuan K., Peng H., Deng Y., Tang Y., Zheng G., Zhou G., Zhao D. (2015). ACS Cent. Sci..

[cit6] Crossland E. J., Noel N., Sivaram V., Leijtens T., Alexander-Webber J. A., Snaith H. J. (2013). Nature.

[cit7] Mao C., Zuo F., Hou Y., Bu X., Feng P. (2014). Angew. Chem., Int. Ed..

[cit8] Yu J., Low J., Xiao W., Zhou P., Jaroniec M. (2014). J. Am. Chem. Soc..

[cit9] Li W., Wu Z., Wang J., Elzatahry A. A., Zhao D. (2013). Chem. Mater..

[cit10] Guan B. Y., Yu L., Li J., Lou X. W. D. (2016). Sci. Adv..

[cit11] Zhang W., Zu L., Kong B., Chen B., He H., Lan K., Liu Y., Yang H., Zhao D. (2018). iScience.

[cit12] Fattakhova-Rohlfing D., Zaleska A., Bein T. (2014). Chem. Rev..

[cit13] Ding D., Liu K., He S., Gao C., Yin Y. (2014). Nano Lett..

[cit14] Xiang Q., Yu J., Jaroniec M. (2012). J. Am. Chem. Soc..

[cit15] Yang Y., Liu G., Irvine J. T., Cheng H. M. (2016). Adv. Mater..

[cit16] Chen X., Burda C. (2008). J. Am. Chem. Soc..

[cit17] Asahi R., Morikawa T., Ohwaki T., Aoki K., Taga Y. (2001). Science.

[cit18] Chen X., Liu L., Peter Y. Y., Mao S. S. (2011). Science.

[cit19] Wang Z., Yang C., Lin T., Yin H., Chen P., Wan D., Xu F., Huang F., Lin J., Xie X., Jiang M. (2013). Energy Environ. Sci..

[cit20] Ohno T., Sarukawa K., Tokieda K., Matsumura M. (2001). J. Catal..

[cit21] Ide Y., Inami N., Saito K., Sohmiya M., Tsunoji N., Komaguchi K., Sano T., Bando Y., Golberg D. (2016). Angew. Chem., Int. Ed..

[cit22] Li A., Wang Z., Yin H., Wang S., Yan P., Huang B., Wang X., Li R., Zong X., Han H., Li C. (2016). Chem. Sci..

[cit23] Koirala R., Pratsinis S. E., Baiker A. (2016). Chem. Soc. Rev..

[cit24] Al-Attafi K., Nattestad A., Wu Q., Ide Y., Yamauchi Y., Dou S. X., Kim J. H. (2018). Chem. Commun..

[cit25] Zhang J., Xu Q., Feng Z., Li M., Li C. (2008). Angew. Chem., Int. Ed..

[cit26] Liu G., Yan X., Chen Z., Wang X., Wang L., Lu G. Q., Cheng H.-M. (2009). J. Mater. Chem..

[cit27] Cao F., Xiong J., Wu F., Liu Q., Shi Z., Yu Y., Wang X., Li L. (2016). ACS Appl. Mater. Interfaces.

[cit28] Kawahara T., Konishi Y., Tada H., Tohge N., Nishii J., Ito S. (2002). Angew. Chem..

[cit29] Yan P., Wang X., Zheng X., Li R., Han J., Shi J., Li A., Gan Y., Li C. (2015). Nano Energy.

[cit30] Linsebigler A. L., Lu G., Yates Jr J. T. (1995). Chem. Rev..

[cit31] Feng X., Shankar K., Varghese O. K., Paulose M., Latempa T. J., Grimes C. A. (2008). Nano Lett..

[cit32] Yang H. G., Sun C. H., Qiao S. Z., Zou J., Liu G., Smith S. C., Cheng H. M., Lu G. Q. (2008). Nature.

[cit33] Cölfen H., Antonietti M. (2005). Angew. Chem., Int. Ed..

[cit34] Ye J., Liu W., Cai J., Chen S., Zhao X., Zhou H., Qi L. (2010). J. Am. Chem. Soc..

[cit35] Elbanna O., Fujitsuka M., Kim S., Majima T. (2018). J. Phys. Chem. C.

[cit36] Li W., Liu J., Zhao D. (2016). Nat. Rev. Mater..

[cit37] Li W., Yang J., Wu Z., Wang J., Li B., Feng S., Deng Y., Zhang F., Zhao D. (2012). J. Am. Chem. Soc..

[cit38] Zhang R., Elzatahry A. A., Al-Deyab S. S., Zhao D. (2012). Nano Today.

[cit39] Joo J. B., Lee I., Dahl M., Moon G. D., Zaera F., Yin Y. (2013). Adv. Funct. Mater..

[cit40] Zhang R., Tu B., Zhao D. (2010). Chem.–Eur. J..

[cit41] Zhou W., Sun F., Pan K., Tian G., Jiang B., Ren Z., Tian C., Fu H. (2011). Adv. Funct. Mater..

[cit42] Tian B., Liu X., Tu B., Yu C., Fan J., Wang L., Xie S., Stucky G. D., Zhao D. (2003). Nat. Mater..

[cit43] Lee J., Orilall M. C., Warren S. C., Kamperman M., DiSalvo F. J., Wiesner U. (2008). Nat. Mater..

[cit44] Yang P., Zhao D., Margolese D. I., Chmelka B. F., Stucky G. D. (1998). Nature.

[cit45] Tan H., Zhao Z., Niu M., Mao C., Cao D., Cheng D., Feng P., Sun Z. (2014). Nanoscale.

[cit46] Zhou W., Li W., Wang J. Q., Qu Y., Yang Y., Xie Y., Zhang K., Wang L., Fu H., Zhao D. (2014). J. Am. Chem. Soc..

[cit47] Li L., Yan J., Wang T., Zhao Z. J., Zhang J., Gong J., Guan N. (2015). Nat. Commun..

[cit48] Liu Y., Che R. C., Chen G., Fan J. W., Sun Z. K., Wu Z. X., Wang M. H., Li B., Wei J., Wei Y., Wang G., Guan G. Z., Elzatahry A. A., Bagabas A. A., Al-Enizi A. M. M., Deng Y. H., Peng H. S., Zhao D. Y. (2015). Sci. Adv..

[cit49] Cheng H., Ma J., Zhao Z., Qi L. (1995). Chem. Mater..

[cit50] Burdett J. K., Hughbanks T., Miller G. J., Richardson Jr J. W., Smith J. V. (1987). J. Am. Chem. Soc..

[cit51] Zhang H., Banfield J. F. (2014). Chem. Rev..

[cit52] Wang G., Wang H., Ling Y., Tang Y., Yang X., Fitzmorris R. C., Wang C., Zhang J. Z., Li Y. (2011). Nano Lett..

[cit53] Jiang X., Zhang Y., Jiang J., Rong Y., Wang Y., Wu Y., Pan C. (2012). J. Phys. Chem. C.

