# Supplementary material for: Synthesis of uniform ordered mesoporous TiO2 microspheres with controllable phase junctions for efficient solar water splitting
Source: Chem Sci. 2018 Nov 21;10(6):1664–70. doi: 10.1039/c8sc04155e (PMC6368211; doi:10.1039/c8sc04155e)
Supplement: Supplementary file 1 [file SC-010-C8SC04155E-s001.pdf]

Electronic Supplementary Material (ESI) for Chemical Science.
This journal is © The Royal Society of Chemistry 2018

Supplementary Information

Synthesis of Uniform Ordered Mesoporous TiO_2 Microspheres with Phase Junctions for Efficient Solar Water Splitting

Wei Zhang,[a] Haili He,[a] Yong Tian,[a] Kun Lan,[a] Qi Liu,[a] Changyao Wang,[a] Yang Liu,[a] Ahmed Elzatahry,[b] Renchao Che,[a] Wei Li,[a] and Dongyuan Zhao[a, *]*

[a]Department of Chemistry, State Key Laboratory of Molecular Engineering of Polymers, Shanghai Key Lab of Molecular Catalysis and Innovative Materials, Laboratory of Advanced Materials, and iChEM, Fudan University, Shanghai 200433, P. R. China
[b] Materials Science and Technology Program, College of Arts and Sciences, Qatar University, PO Box 2713, Doha 2713, Qatard
* Corresponding author.
E-mail: weilichem@fudan.edu.cn(W.Li); dyzhao@fudan.edu.cn (D. Y. Zhao).

Keywords: Mesoporous materials; TiO_2 phase junctions; Water splitting.

Experimental section

Chemicals

Experimental Section

Chemicals. Anhydrous ethanol, methanol, tetrahydrofuran (THF), concentrated hydrochloric acid (HCl), acetic acid (HOAc) and chloroplatinic acid hexahydrate ($H_2PtCl_6{\cdot}6H_2O$) were obtained from Sinopharm Chemical Reagent Co., Ltd. (Shanghai, China). Tetrabutyl titanate (TBOT) was obtained from Aladdin Chemical Reagent Co., Ltd. Amphiphilic triblock copolymer Pluronic F127 (PEO_{106}-PPO_{70}-PEO_{106}) was purchased from Alfa Aesar. Commercial TiO_2 P25 was purchased from Evonik Co., Ltd. Deionized water (Millipore) was used in all experiments.

Synthesis of Meso-TiO_2-25. The Meso-TiO_2-25 were synthesized by a coordination-mediated self-assembly strategy. Typically, 1.5 g of Pluronic F127 was mixed with 2.0 g of HCl, 2.0 g of HOAc, 3.4 g of TBOT and 30 ml of THF to form a clear and transparent yellow solution. After that, the resultant solution was transferred into a volumetric flask, and then left it in a drying oven to evaporate the solvents at 40 °C for 20 h and at 80 °C for another 8 h to form a white powder. Finally, the Meso-TiO_2-25 were obtained by calcination of the white powder in nitrogen (N_2) and air at 400 °C for 3 h, respectively. The mesoporous TiO_2 microspheres with different anatase to rutile ratios (the percentage of rutile from 0 to 100) could be obtained by facilely adjusting the usage of HCl.

Photoelectrochemical properties. Photocurrents were measured in a three-electrode system, in which the ordered mesoporous TiO_2 microspheres (~ 10 mg) film on a fluorine-doped tin oxide (FTO) substrate (2*2 cm^2), a Pt wire, and an Ag/AgCl were used as the working, count, and reference electrodes, respectively. 1.0 M KOH aqueous solution was used as an electrolyte to maintain the stability of films. A Xe lamp (300 W, Perfectlight Co., Ltd.) with a power of 100 mW cm^{-2} was used as the illumination source. Linear sweep voltammetry was carried out on a CHI660e (CH instruments Co., Ltd.) workstation. Photocurrent ON/OFF cycles were measured using the same electrochemical workstation coupled with a mechanical chopper.

Photocatalytic H_2 generation. Photocatalytic hydrogen generation was measured using an online photocatalytic hydrogen generation system (Labsolar-6A, Perfectlight Co., Ltd.) at an ambient temperature (25 °C). 50 mg of photocatalysts loaded with 1 wt% Pt was added to an aqueous solution (75 ml of H_2O, 25 ml of methanol) in a closed gas circulation system. Before irradiation, the reactor and the whole gas circulating system were fully degassed to remove air by using a vacuum pump for 30 min. The amount of H_2 generated was determined by gas chromatography (Techcomp 7900, TCD, N_2 carrier). A Xe lamp (300 W, Perfectlight Co., Ltd.) with an optical filter was used as the illumination source.

Photocatalytic Degradation Activity Measurement: The photocatalytic degradation of rhodamine B (RhB) was carried out in an aqueous solution at room temperature under UV-visible light irradiation. A Xe lamp (300 W, Perfectlight Co., Ltd.) with an optical filter (AM 1.5G) was used as the illumination source. The reactor was open to air in order to reach the air-equilibrated condition. In a typical experiment, 50 mg of the photocatalyst powders was dispersed in 50 mL of rhodamine B (1×10^{-4} M) with stirring. The suspension was stirred for 12 h in the dark to reach adsorption equilibrium. At given time intervals, 3.0 mL of aliquots was collected, centrifuged, and then filtered to remove the catalyst particles for analysis. The filtrates were finally analyzed by an UV–vis spectrophotometer (Lambda 650S UV).

Characterizations. X-ray diffraction (XRD) patterns were collected by a Bruker D8 powder X-ray diffractometer (Germany) with Ni-filtered Cu Kα radiation (40 kV, 40 mA). Field-emission scanning electron microscopy (FESEM) images were obtained on a Hitachi S4800 (Japan) operated at 1 kV and 10 μA. Transmission electron microscope (TEM) measurements were conducted on a JEOL 2100F microscope (Japan) operated at 200 kV. The samples for the TEM measurements were suspended in ethanol and supported onto a holey carbon film on a Cu grid. N_2 adsorption-desorption isotherms were measured with a Micromeritics Tristar 2420 analyzer at 77 K. Before measurements, the samples were degassed at 180 °C for 6 h. The

Brunauer-Emmett-Teller (BET) method was utilized to calculate the specific surface areas. The pore size distributions were derived from the adsorption branches of the isotherms based on the Barrett-Joyner-Halenda (BJH) modal. The total pore volumes were estimated from the amount adsorbed at a relative pressure (P/P_0) of 0.99. X-ray photoelectron spectroscopy (XPS) was recorded on a RBD upgraded PHI-5000C ESCA system (Perkin Elmer) with Mg Kα radiation ($h\nu$ = 1253.6 eV). Ultraviolet-visible (UV-Vis) diffuse reflectance spectra were collected on Lambda 650S UV spectrophotometer with the wavelength from 200 to 800 nm. Raman spectra were performed with a Horiba Jobin Yvon XploRA spectrometer at 457.9 nm, the Kaser beam was focused with a 50 × objective lens to a *ca*. 1 μm spot on the surface of samples. Fluorescence spectroscopy were recorded on Edinburgh Fluorescence Spectrometer FLS980 instrument with the excitation light at 260 nm. Scanning Kelvin probe (SKP) tests (SKP5050 system, Scotland) were executed to evaluate the surface work functions at ambient atmosphere.

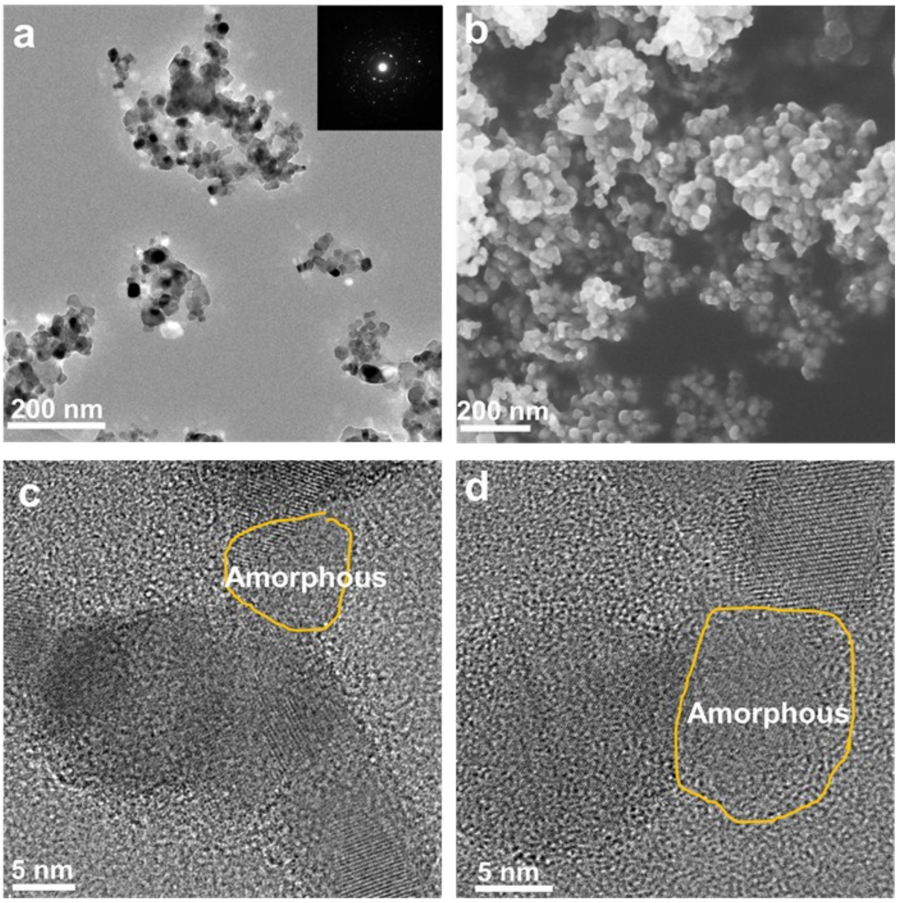

Figure S1. TEM (a), FESEM (b) and HRTEM (c-d) images of commercial P25 nanoparticles purchased from Evonik Company. The inset in (a) is the corresponding SAED pattern.

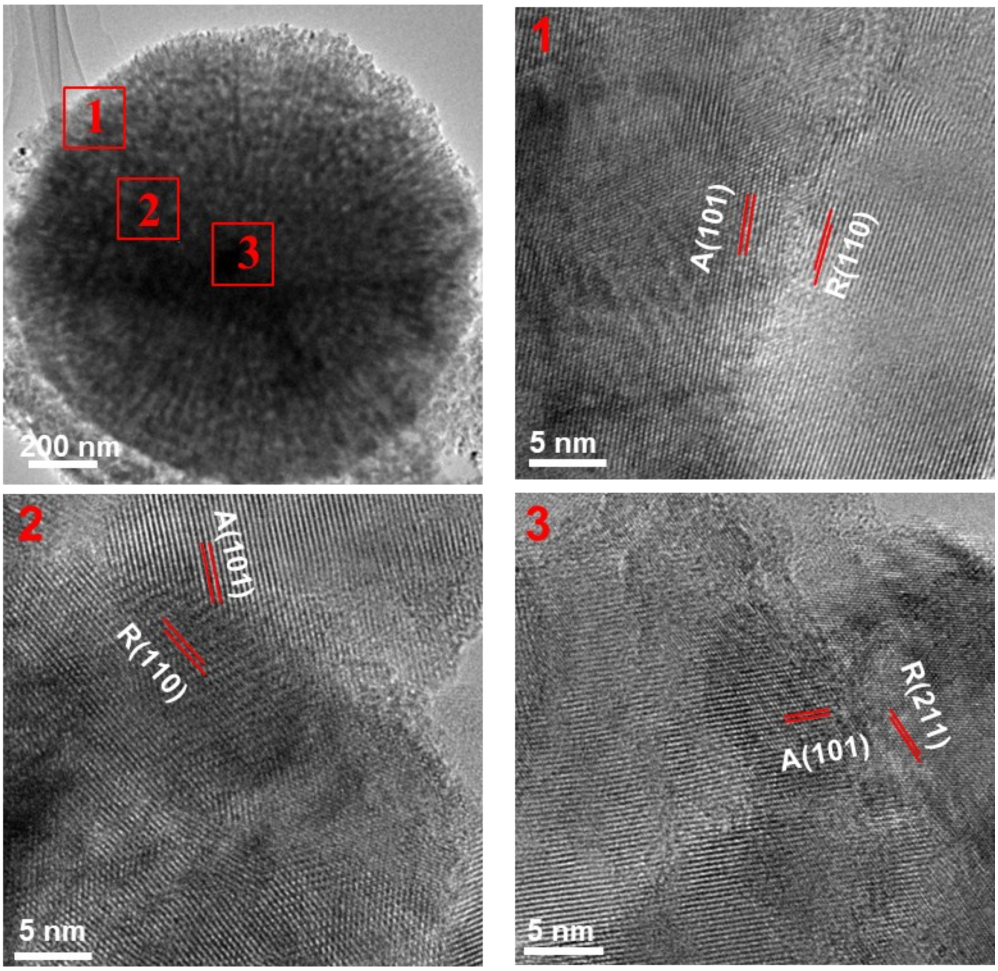

Figure S2. TEM images of three representative interfacial domains taken from one single Meso-TiO_2-25 microsphere from outside to inside to demonstrate the formation of phase junctions. Before the TEM characterization, the Meso-TiO_2-25 were embedded in a resin and cut into microtomed slices.

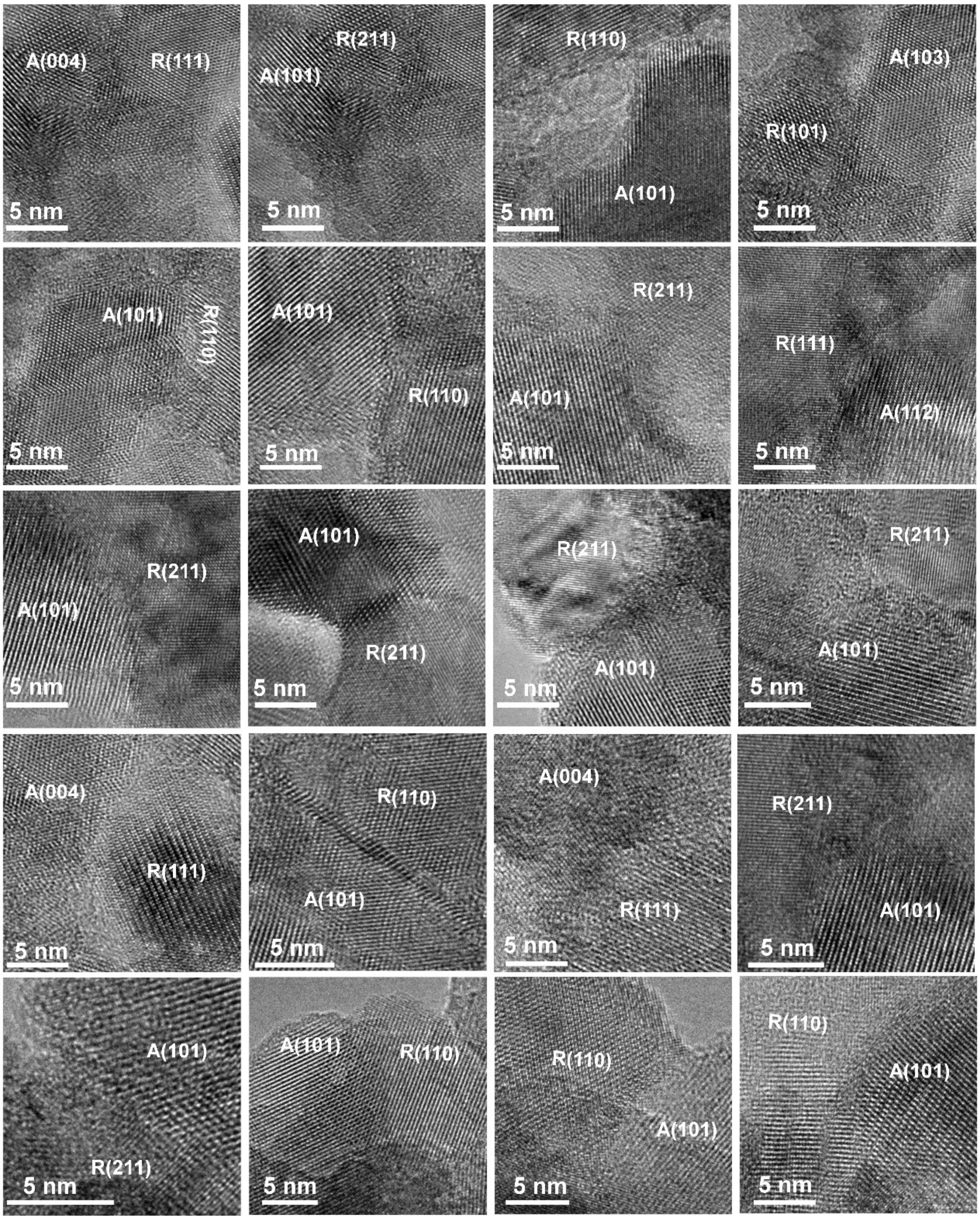

Figure S3. Randomly selected HRTEM images of the Meso-TiO_2-25, showing the directly contact anatase-rutile phase junctions. These images clearly indicate that the nanocrystals are randomly oriented, but the directly contact anatase and rutile nanoparticles are lattice-matching.

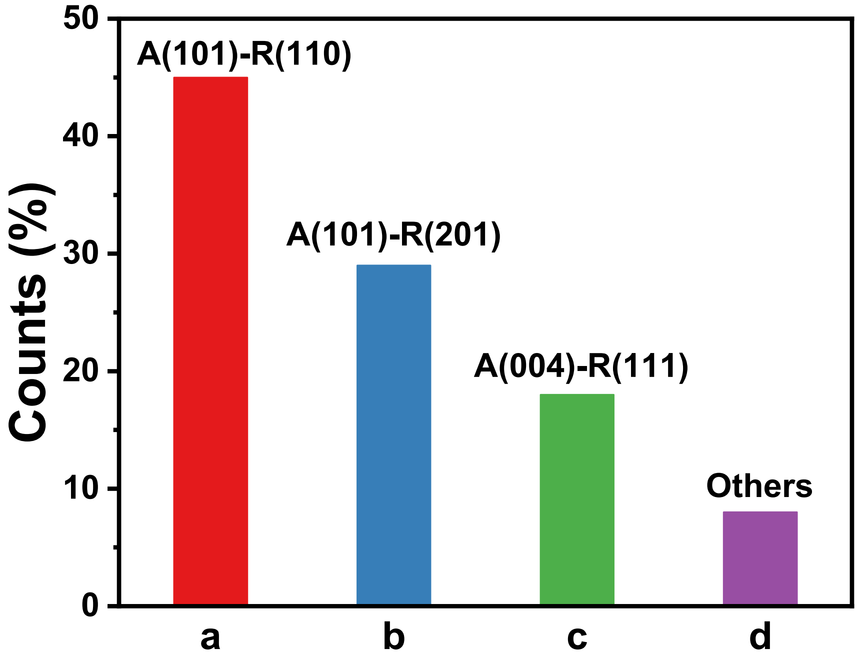

Figure S4. The statistic results of the exposed crystal faces between intimate contact anatase and rutile nanoparticles in the Meso-TiO_2-25 based on 100 phase junctions, indicating that the lattice-matched phase junctions have many possibilities.

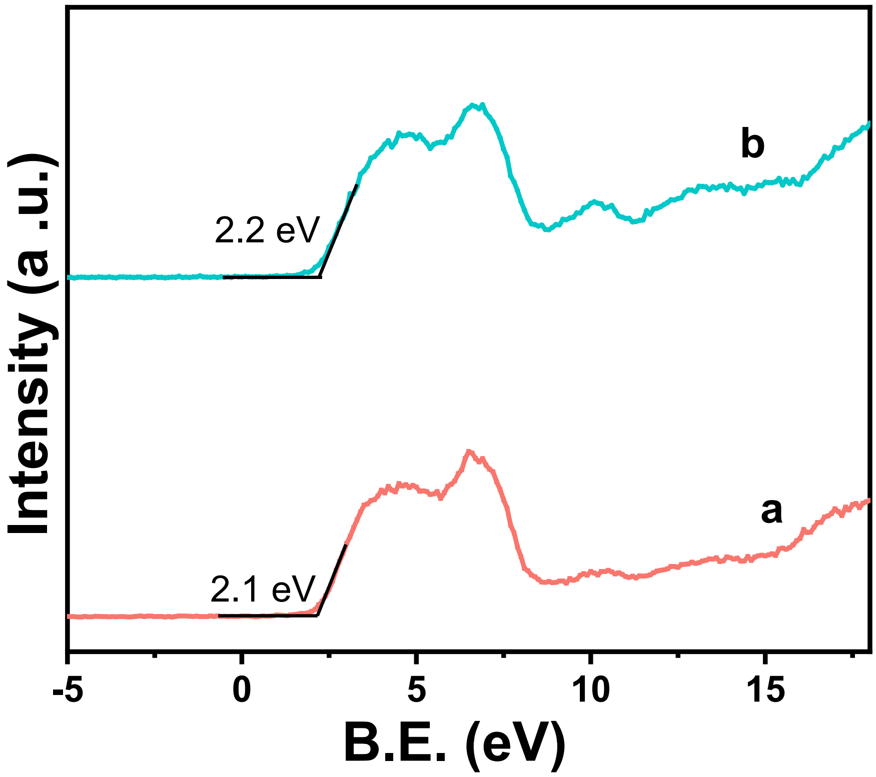

Figure S5. Valence band XPS characteristic curves for the Meso-TiO_2-25 (a) and commercial P25 (b).

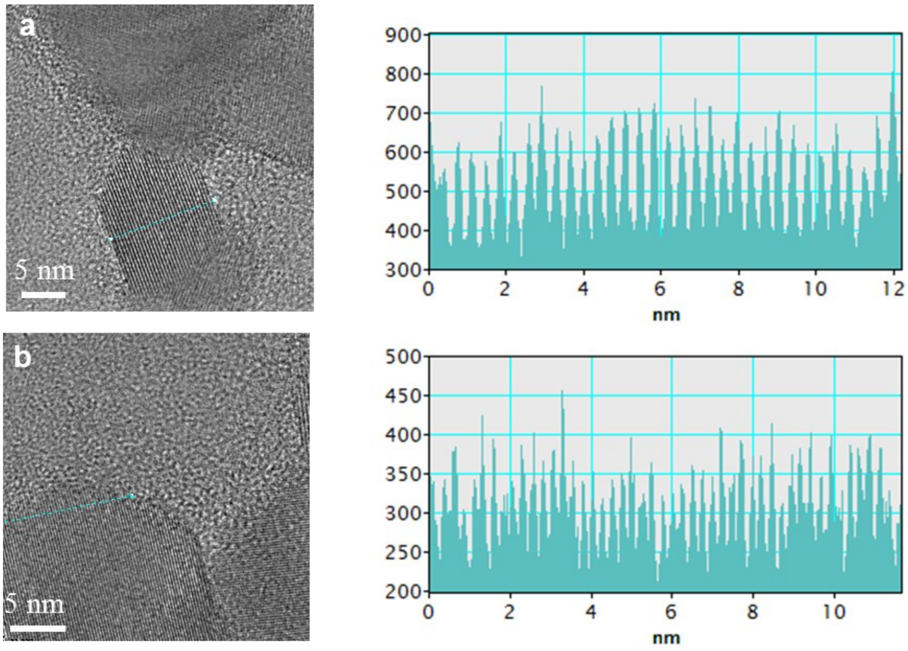

Figure S6. HRTEM images of the commercial P25 and the corresponding structural analysis. The analysis region is marked in the HRTEM images.

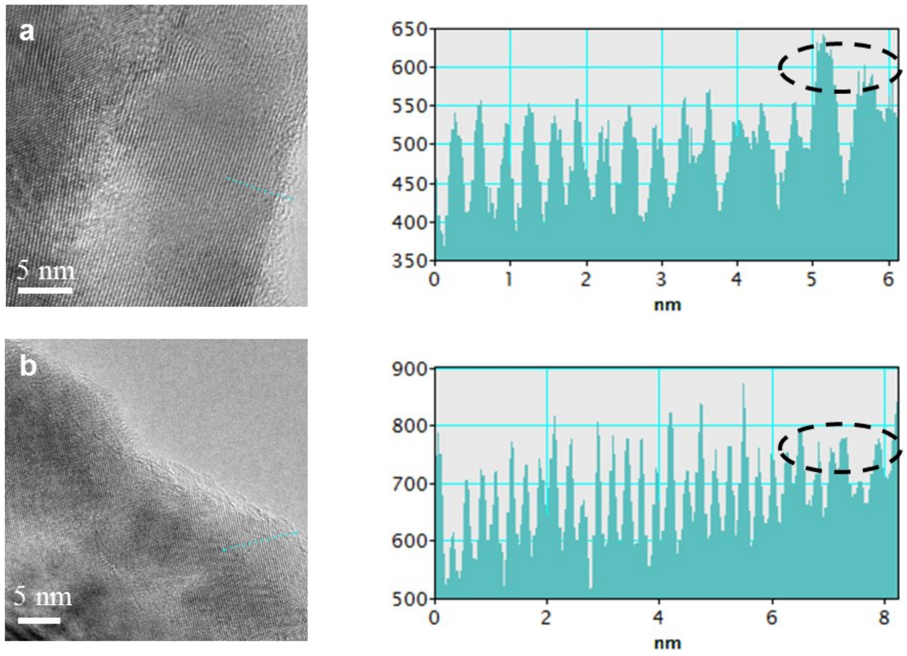

Figure S7. HRTEM images of the Meso-TiO_2-25 and the corresponding structural analysis, indicating that the surface defects are formed during the sol-gel process. The analysis region is marked in the HRTEM images.

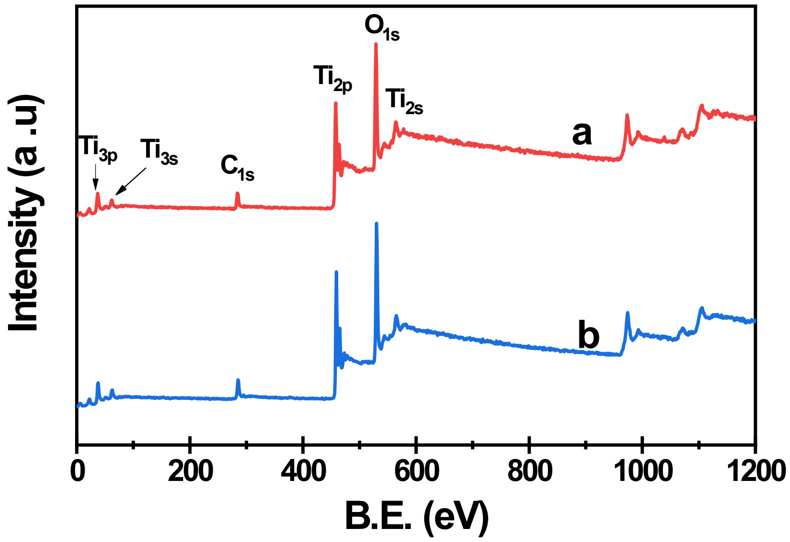

Figure S8. XPS survey spectra of the Meso-TiO_2-25 (a) and commercial P25(b).

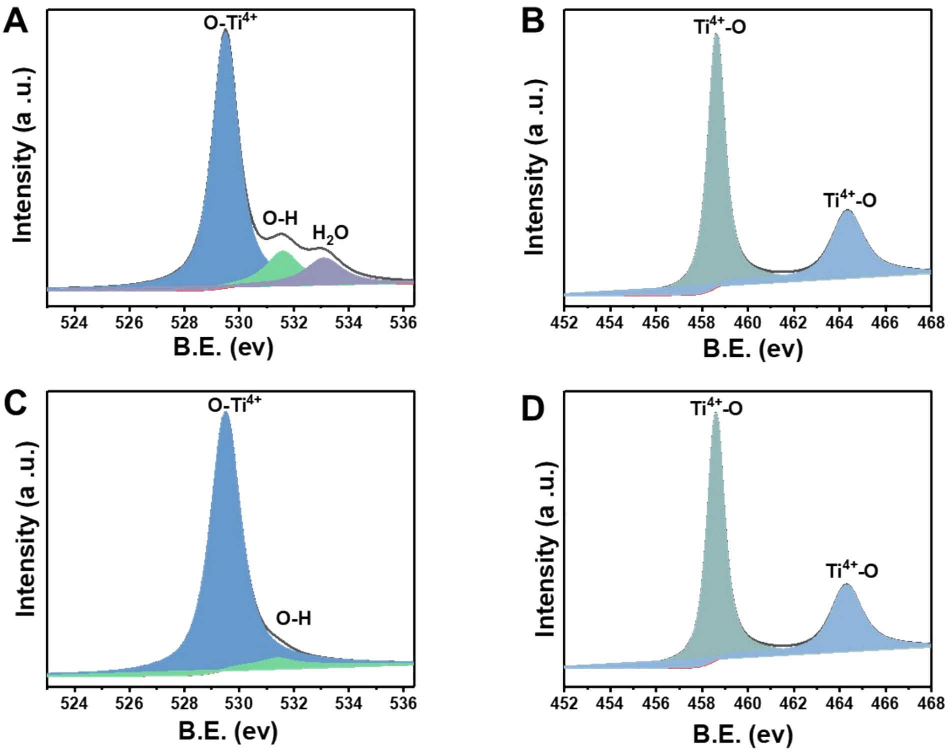

Figure S9. High-resolution XPS spectra of Ti_{2p} (A, C) and O_{1s} (B, D) for the Meso-TiO_2-25 (A, B) and commercial P25 (C, D).

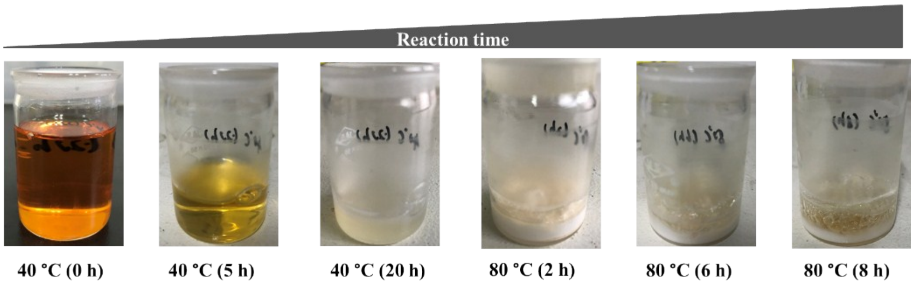

Figure S10. Photographs of the intermediate products taken from different reaction time during the solvent evaporation process. After the evaporation at 40 °C for 20 h, the clear solution was transformed to a gel. After further soaking the gel at a high temperature (80 °C), white powders could be obtained.

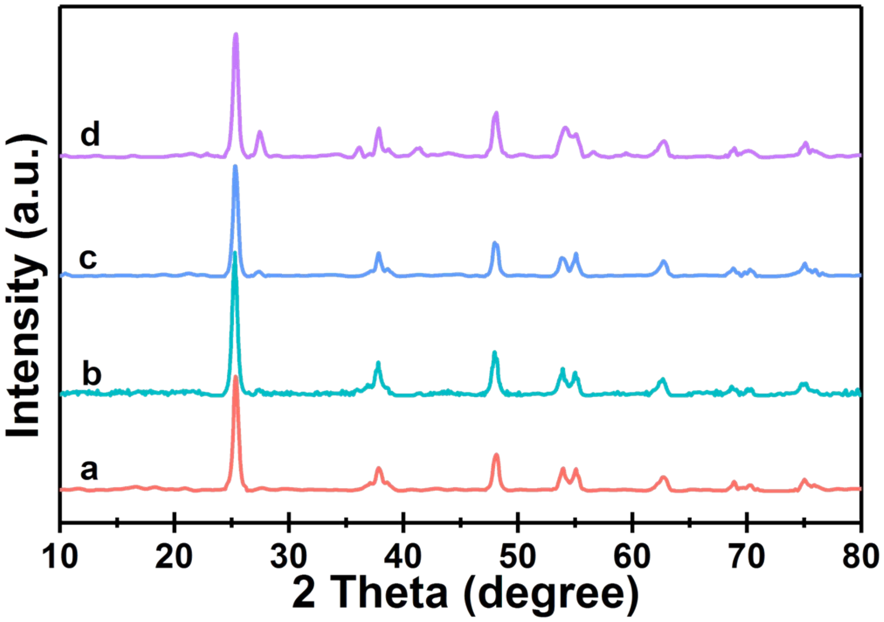

Figure S11. XRD patterns the intermediate products directly taken from different reaction time during the evaporation process, (a) 40 °C, for 20 h, (b) 80 °C, for 2 h, (c) 80 °C, for 4 h, (d) 80 °C, for 8 h. All samples were annealed in N_2 and air at 400 °C for 3 h, respectively.

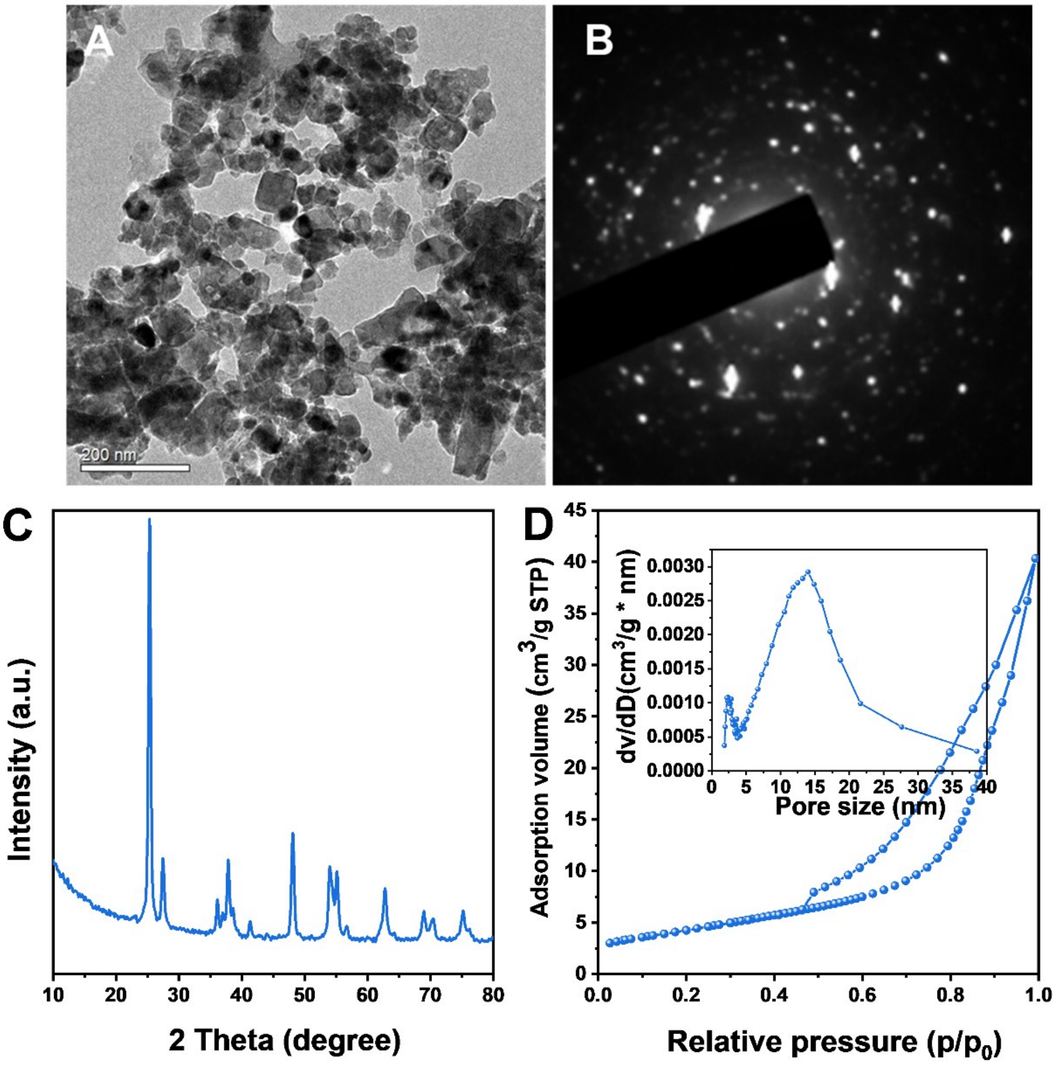

Figure S12. TEM (A), SAED (B), XRD (C) and nitrogen adsorption-desorption isotherms (D) of nonporous-TiO_2-25 synthesized without using Pluronic F127. The inset in (D) is the corresponding pore size distribution curve.

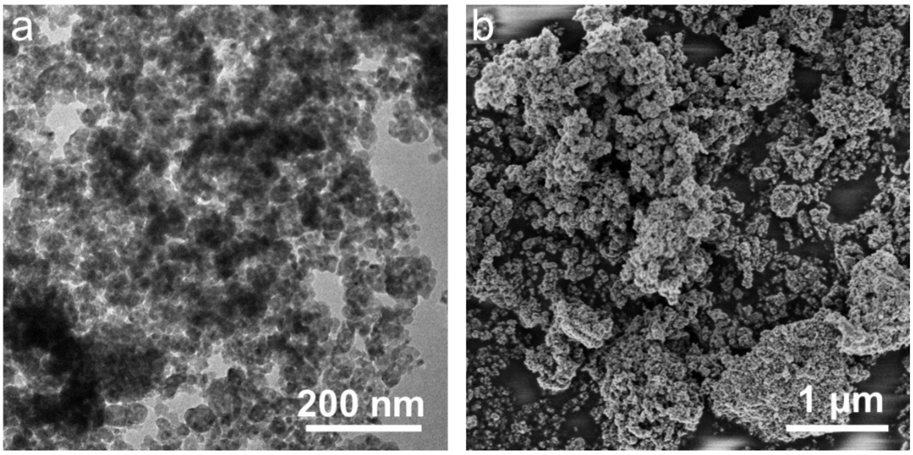

Figure S13. TEM (a) and FESEM (b) images of the sample synthesized without using HOAc, indicating that HOAc plays a key role on the formation of spherical mesostructure.

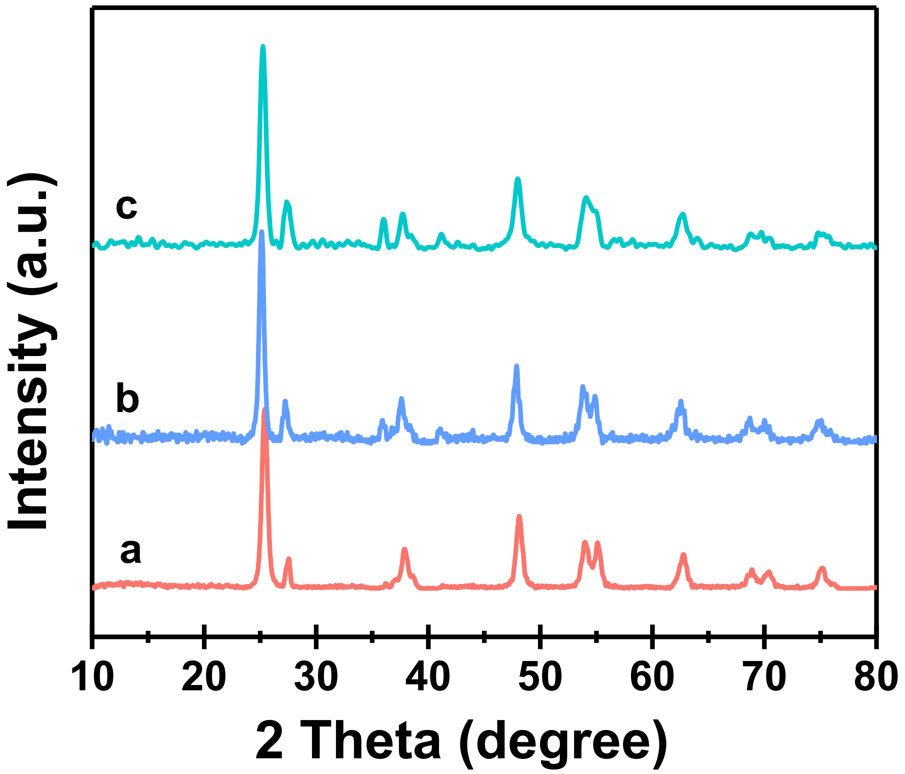

Figure S14. XRD patterns of the ordered mesoporous TiO_2 microspheres prepared with different concentration of HOAc: (a) 0.5 M, (b) 1.0 M, and (c) 1.5 M. All samples were annealed in N_2 and air at 400 °C for 3 h, respectively.

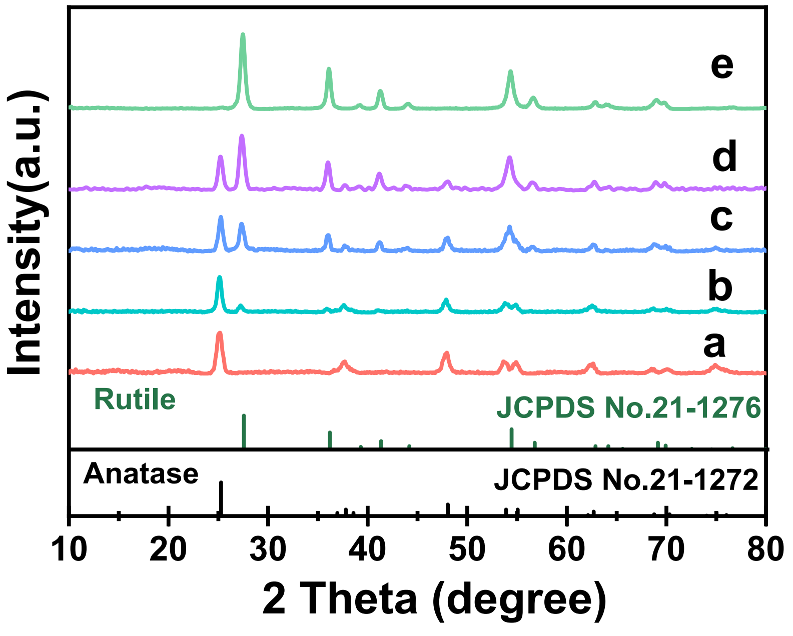

Figure S15. XRD patterns of the ordered mesoporous TiO_2 microspheres with different anatase /rutile ratios synthesized by the seed-mediated self-assembly method through changing the usage of HCl. (a) 0.3 M, (b) 0.6 M, (c) 0.9 M, (d) 1.2 M and (e) 1.5 M. All samples were annealed in N_2 and air at 400 °C for 3 h, respectively.

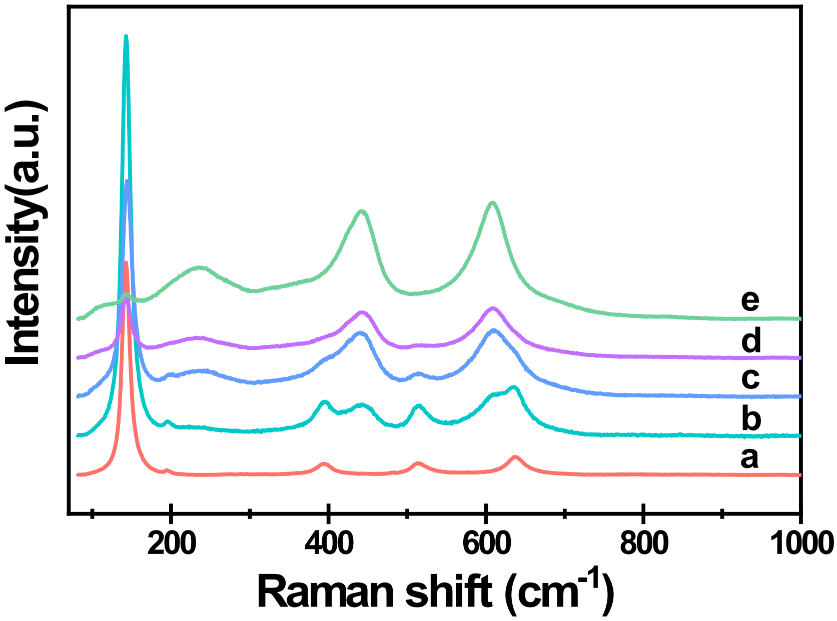

Figure S16. Raman spectra of the ordered mesoporous TiO_2 microspheres with different anatase/rutile ratios synthesized by the seed-mediated self-assembly method through changing the usage of HCl. (a) 0.3 M, (b) 0.6 M, (c) 0.9 M, (d) 1.2 M and (e) 1.5 M. All samples were annealed in N_2 and air at 400 °C for 3 h, respectively.

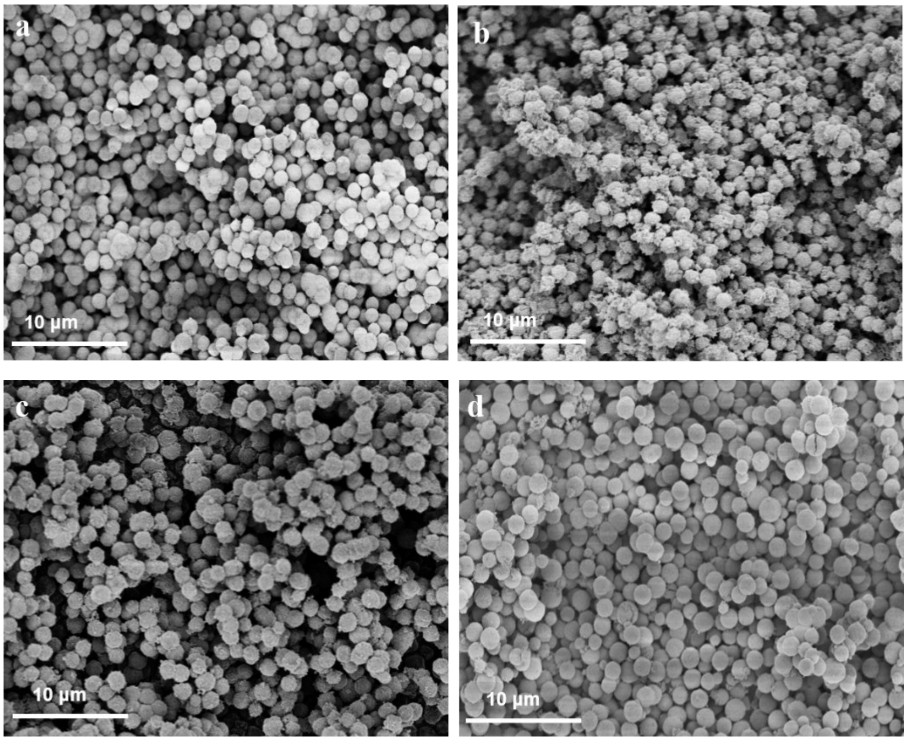

Figure S17. FESEM images of (a) Meso-TiO_2-0, (b) Meso-TiO_2-40, (c) Meso-TiO_2-60 and (d) Meso-TiO_2-100 microspheres.

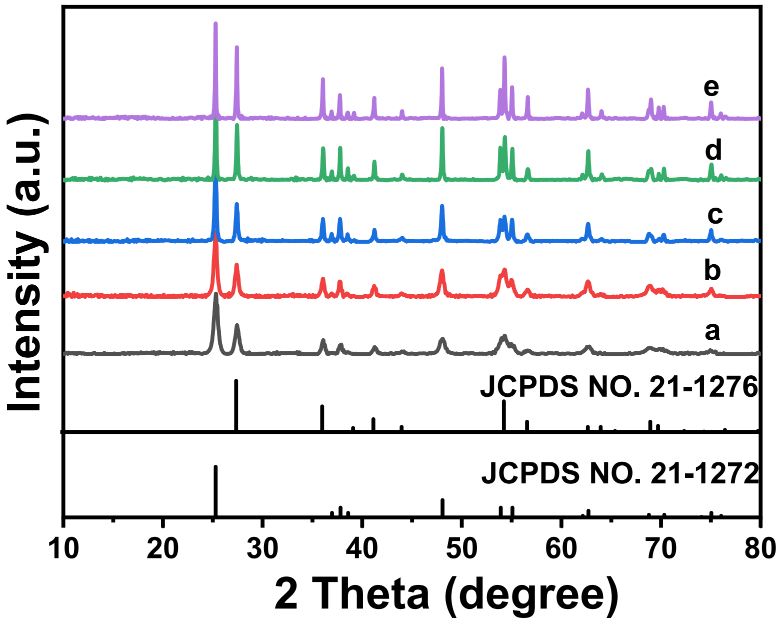

Figure S18. XRD patterns of the ordered mesoporous TiO_2 microspheres calcinated at different temperatures in air. (a) 500 °C, (b) 600 °C, (c) 700 °C, (d) 800 °C and (e) 900 °C.

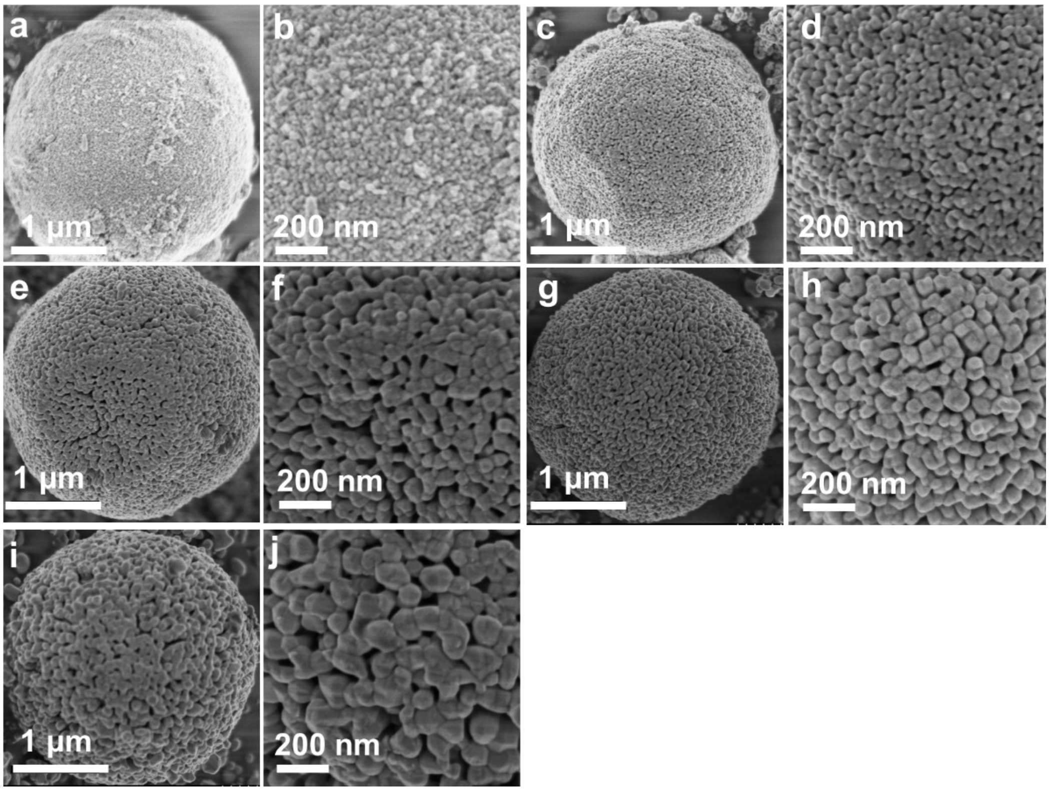

Figure S19. SEM images of the ordered mesoporous TiO_2 microspheres calcinated at different temperatures in air. (a, b) 500 °C, (c, d) 600 °C, (e, f) 700 °C, (g, h) 800 °C and (i, j) 900 °C.

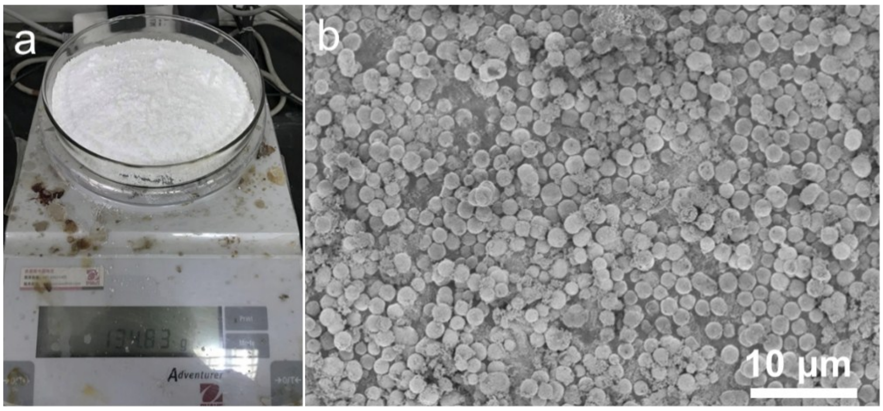

Figure S20. The photograph (a) of the large-scale production for the Meso-TiO_2-25 synthesized by the coordination-mediated self-assembly in one-pot and the corresponding FESEM image (b).

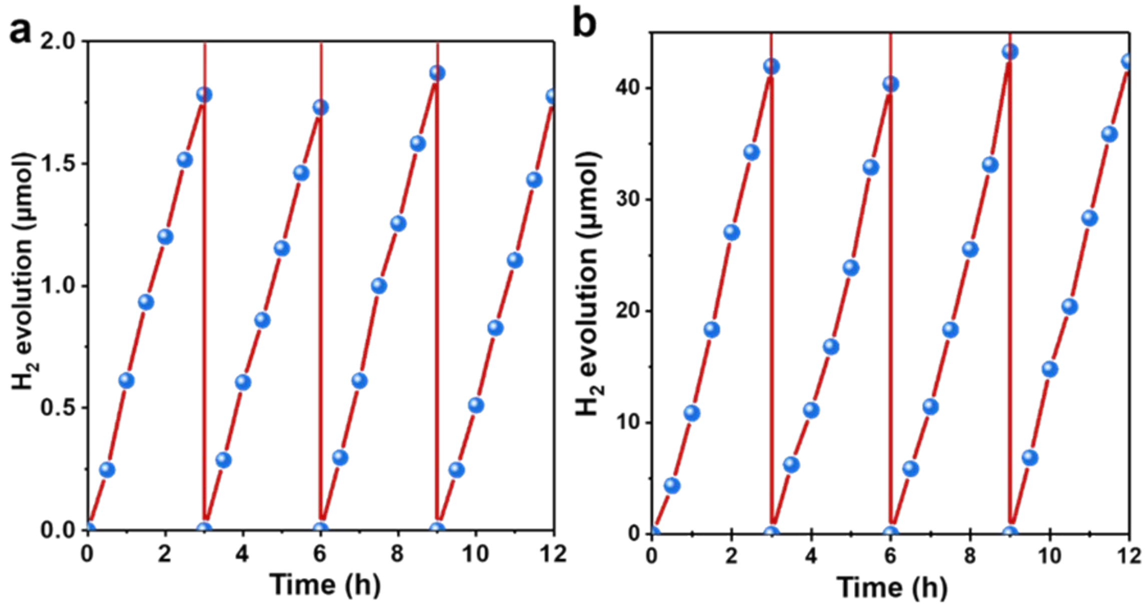

Figure S21. The cyclic stability of the Meso-TiO_2-25 for photocatalytic H_2 generation under AM 1.5G (a) and visible-light of λ > 400 nm (b), respectively (The reactor was degassed in vacuum before each cycling test)

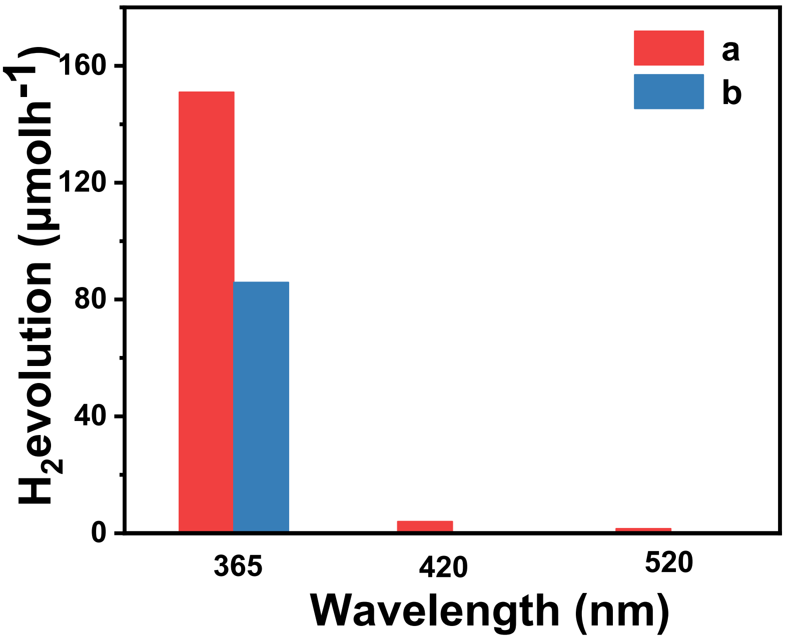

Figure S22. The H_2 generation rate of (a) the Meso-TiO_2-25 microspheres and (b) commercial P25 under single-wavelength light (based on 50 mg of catalysts).

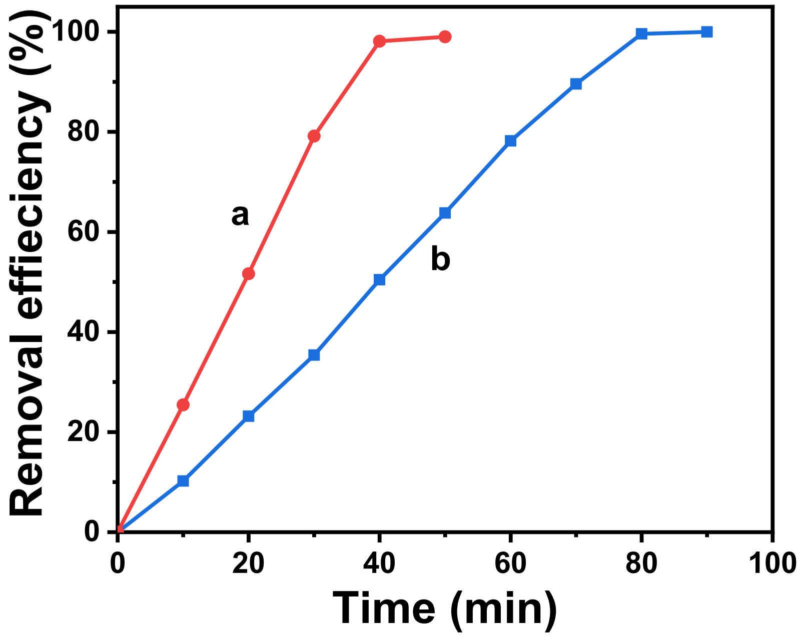

Figure S23. Photocatalytic degradation curves of RhB under a simulated sunlight light irradiation using (a) Meso-TiO_2-25 microspheres and (b) commercial P25.

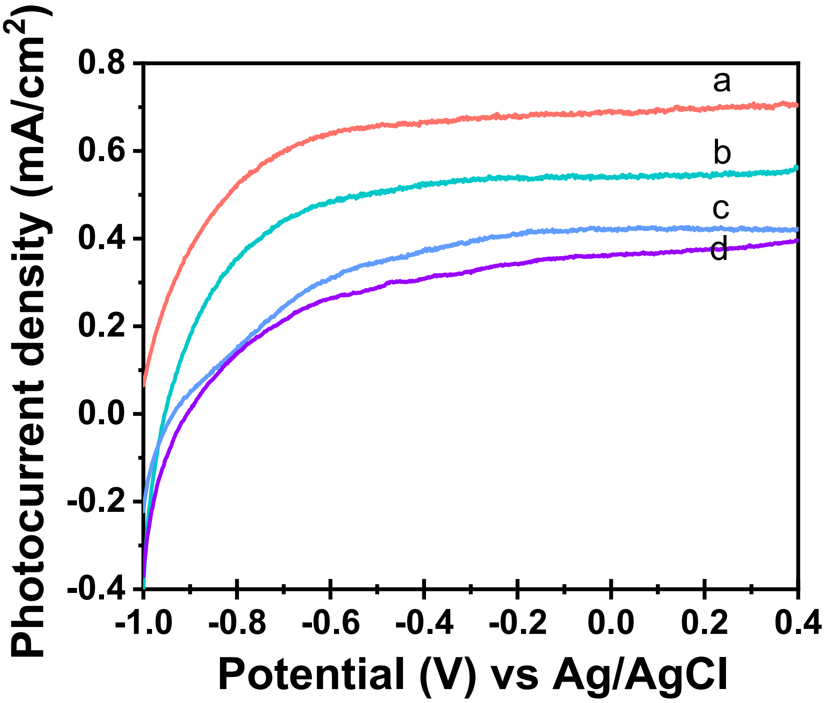

Figure S24. Photocurrent densities of the Meso-TiO_2-25 (a), Meso-TiO_2-0 (b), Meso-TiO_2-100 (c) and commercial P25 (d).

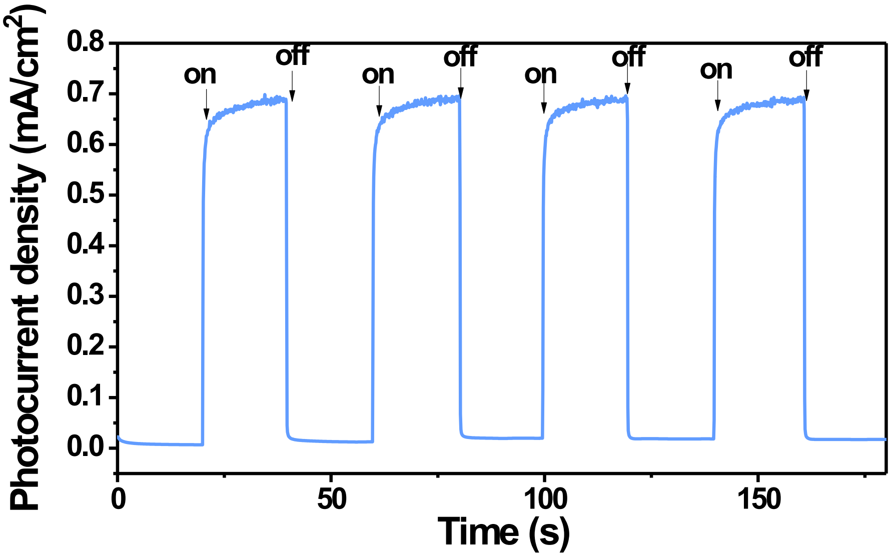

Figure S25. Time-dependent photocurrent density of the Meso-TiO_2-25 measured at 0.2 V *vs* Ag/AgCl with repeated on/off cycles of simulated sunlight illumination.

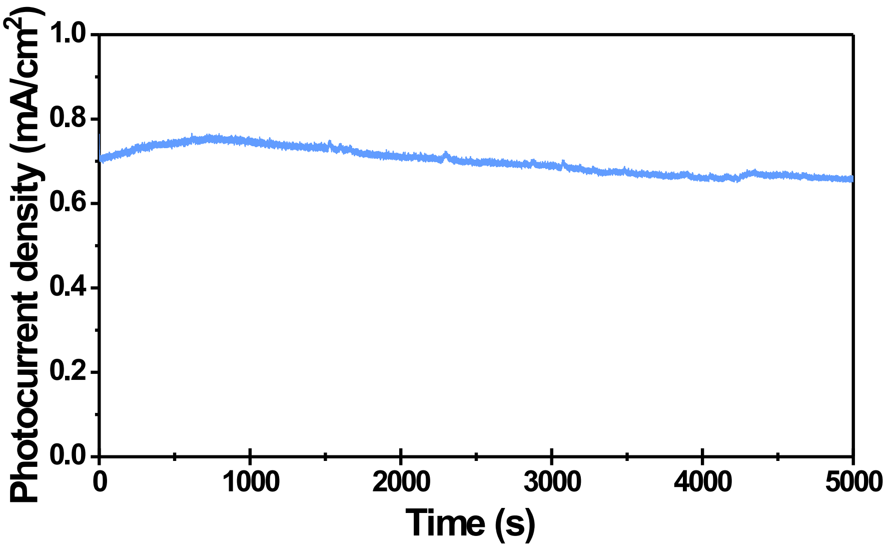

Figure S26. Photocurrent density of the Meso-TiO_2-25 measured at 0.2 V *vs* Ag/AgCl under continuous solar illumination for 5000 s.

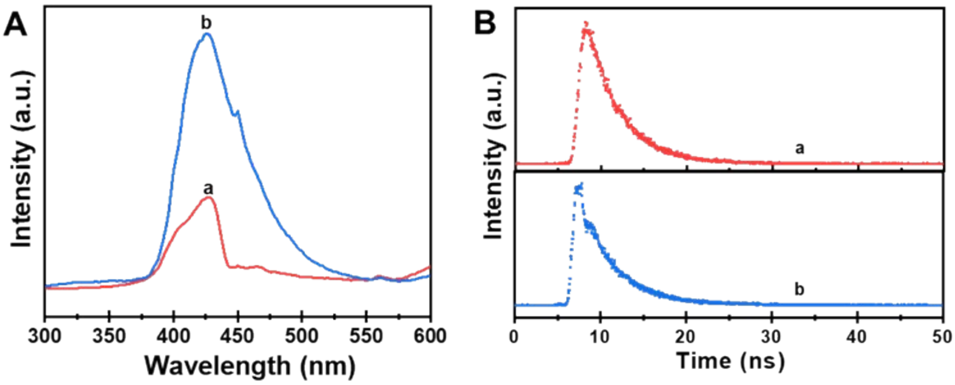

Figure S27. (A) The fluorescence spectroscopy and (B) time-resolved fluorescence spectroscopy for (a) Meso-TiO_2-25, (b) commercial P25. The decay time of Meso-TiO_2-25 and (b) commercial P25 are measured to be 3.82 and 3.70 ns, respectively.

Table S1. Comparison of photocatalytic activities in hydrogen production from water splitting over TiO_2-based photocatalysts.

Photocatalyst	Incident light	Reactant solution	Co-catalyst	H_2 evolution rate (μmol g^{-1} h^{-1})	Ref.
Hydrogenated H-TiO_2	AM 1.5G<br>λ >400 nm	50 % CH_3OH/H_2O	0.6% Pt	10000<br>100	(1)
Hydrogenated titanate naotube	AM 1.5G<br>λ >400 nm	20 % CH_3OH/H_2O	1% Pt	2150<br>120	(2)
Al reduced H-TiO_2	AM 1.5G<br>λ >400 nm	25 % CH_3OH/H_2O	0.5% Pt	6400<br>140	(3)
S-doped H-TiO_2	AM 1.5G	25 % CH_3OH/H_2O	0.5% Pt	258	(4)
N-doped H-TiO_2	AM 1.5G<br>λ >400 nm	20 % CH_3OH/H_2O	0.5% Pt	15000<br>200	(5)
Mg reduced H-TiO_2	Xe lamp, 400 W<br>λ >400 nm	20 % CH_3OH/H_2O	1% Pt	43200<br>440	(6)
Ti^{3+} self-doped TiO_2	λ >400 nm	25 % CH_3OH/H_2O	1% Pt	181	(7)
Sub-10 nm rutile TiO_2 Nanoparticles	AM 1.5G<br>λ >400 nm	10 % CH_3OH/H_2O	1% Pt	1954<br>932	(8)
vacuum activated TiO_2 P25	λ >400 nm	25 % CH_3OH/H_2O	0.38% Pt	120	(9)
TiO_2 with oxygen Vacancies	λ >400 nm	25 % CH_3OH/H_2O	1% Pt	115	(10)
Ordered Meso-TiO_2-25 microspheres	AM 1.5G<br>λ >400 nm	25 % CH_3OH/H_2O	1% Pt	12600<br>293	This work

References

(1) X. Chen, L. Liu, P. Y. Yu, S. S. Mao, *Science,* 2011, **331**, 746.

(2) Z. Zheng, B. Huang, J. Lu, Z. Wang, X. Qin, X. Zhang, M. H. Whangbo, *Chem. Commun*, 2012, **48**, 5733.

(3) Z. Wang, C. Yang, T. Lin, H. Yin, P. Chen, D. Wan, F. Xu, F. Huang, J. Lin, X. Xie, M. Jiang, *Energy Environ. Sci.* 2013, **6**, 3007.

(4) C. Yang, Z. Wang, T. Lin, H. Yin, X.Lu, D. Wan, T. Xu, C. Zheng, J. Lin, F. Huang, X. Xie, M. Jiang, *J. Am. Chem. Soc*. 2013, **135**, 17831.

(5) T. Lin, C. Yang, Z. Wang, H. Yin, X. Lv, F. Huang, J. Lin, X. Xie, M. Jiang, *Energy Environ. Sci.* 2014, **7**, 967.

(6) A. Sinhamahapatra, J. P. Jeon, J. S. Yu, *Energy Environ. Sci.* 2015, **8**, 3539.

(7) F. Zuo, R. J. Dillon, L. Wang, P. Smith, X. Zhao, P. Feng, *Angew. Chem. Int. Ed.* 2012, **124**, 6223.

(8) L. Li, J. Yan, T. Wang, Z. J. Zhao, J. Zhang, J. Gong, N. Guan, *Nat. Commun.* 2015, **6**, 5881.

(9) M. Xing, J. Zhang, F. Chen, B. Tian, *Chem. Commun.* 2011, **47**, 4947.

(10) X. Zou, J. Liu, J. Su, F. Zuo, J. Chen, P. Feng, *Chem. Eur. J.* 2013, **19**, 2866.
